# Distinctive features of cancer-associated fibroblasts expressing CD105, a novel biomarker for bone metastasis, in early-stage invasive ductal breast cancer

**DOI:** 10.3389/fendo.2026.1766643

**Published:** 2026-02-20

**Authors:** María Belén Giorello, Francisco Raúl Borzone, María Cecilia Sanmartin, Leandro Marcelo Martinez, Mrinmoy Sarkar, Tapasree Roy Sarkar, Vivian Labovsky, Alejandra Wernicke, Norma Alejandra Chasseing

**Affiliations:** 1 Laboratorio de Inmunohematología, Instituto de Biología y Medicina Experimental (IBYME), Fundación IBYME, Consejo Nacional de Investigaciones Científicas y Técnicas (CONICET), Ciudad Autónoma de Buenos Aires, Buenos Aires, Argentina; 2 Laboratorio de Medicina Regenerativa Cardiovascular, Instituto de Medicina Traslacional, Trasplante y Bioingeniería (IMETTyB-Universidad Favaloro-CONICET), Ciudad Autónoma de Buenos Aires, Buenos Aires, Argentina; 3 Division of Hematology and Medical Oncology, Department of Medicine, Weill Cornell Medical College, New York, NY, United States; 4 Department of Biology, Faculty, Genetics and Genomics, Center for Biological Clock Research (CBCR) Texas A&M University, College, Station, TX, United States; 5 Departamento de Anatomía Patológica, Hospital Italiano, Ciudad Autónoma de Buenos Aires, Buenos Aires, Argentina

**Keywords:** breast cancer, cancer-associated fibroblasts, CD105, metastasis, osteoblast-like cancer cells, stemness genes induction

## Abstract

**Purpose:**

Cancer-associated fibroblasts (CAFs) are highly heterogeneous and critically influence breast cancer progression, yet functionally relevant stromal subpopulations remain poorly defined. This study investigates whether CD105 expression distinguishes fibroblast subsets with distinct mesenchymal stem–like properties and tumor-modulating functions within early-stage breast cancer.

**Methods:**

CD105(+)/CD34(-) and CD105(-)/CD34(-) fibroblast subpopulations were isolated from primary luminal breast tumors and analyzed for phenotypic, molecular, and functional characteristics, as well as for their effects on breast cancer cell behavior using *in vitro* assays, including conditioned media (CM) approaches.

**Results:**

Both fibroblast subpopulations displayed mesenchymal stem/stromal cell (MSC) characteristics; however, CD105(+)/CD34(-) fibroblasts displayed a more pronounced MSC–like phenotype, with enhanced proliferative capacity, altered oxidative status, and a distinct gene expression and secretory profile. CM from CD105(+)/CD34(-) fibroblasts more strongly promoted migration and proliferation and increased the expression of genes associated with stemness, osteogenic differentiation, bone mineralization, and osteoclastogenesis in luminal and triple-negative human breast cancer cell lines, compared with CM from CD105(-)/CD34(-) fibroblasts.

**Conclusion:**

These findings identify CD105 as a potential functional discriminator of breast CAF subpopulations and suggest that CD105(+) fibroblasts may preferentially support tumor progression and the acquisition of bone-related traits. This work provides new insight into CAF heterogeneity and its potential relevance for metastatic progression, and may also guide future therapeutic strategies and research directions.

## Graphical Abstract

**Figure:**
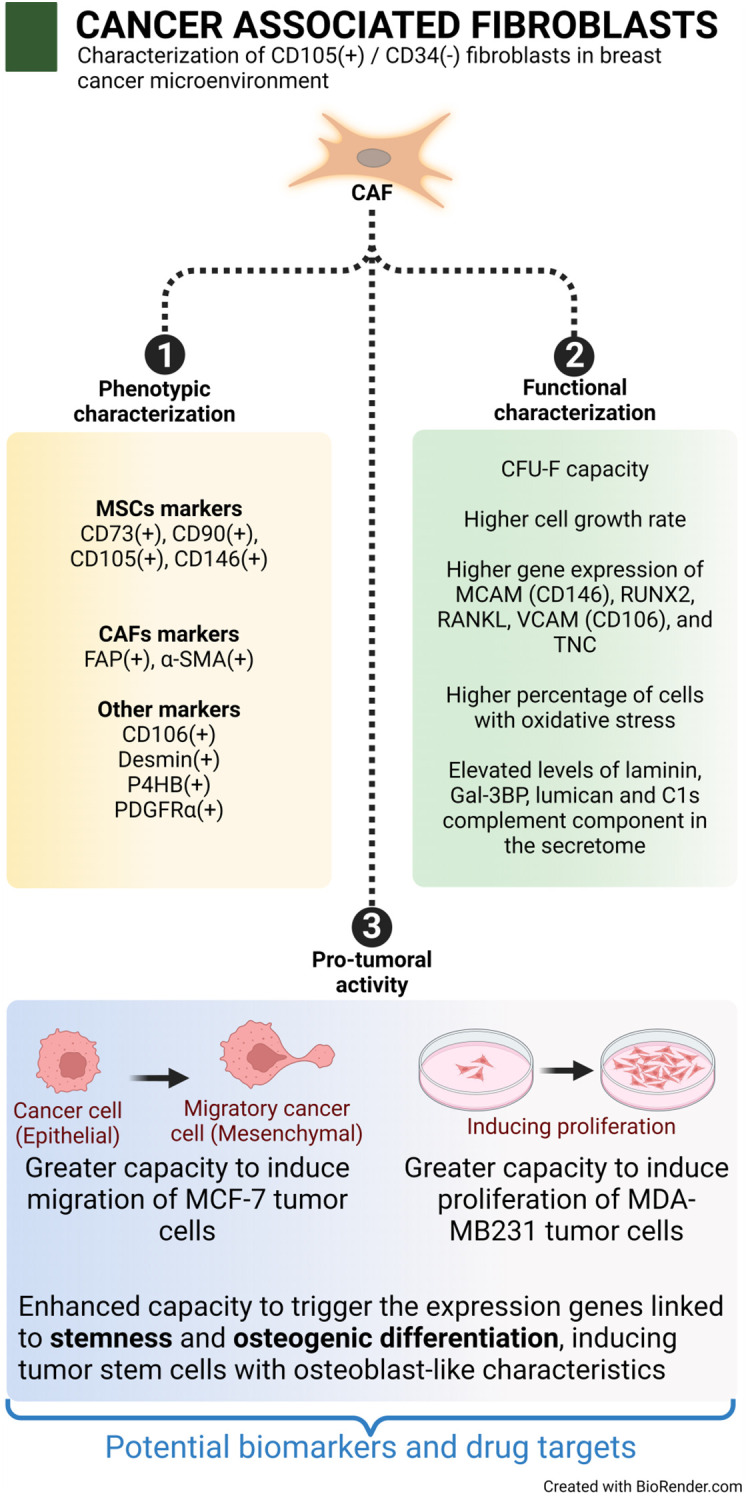
Summary of the key findings of the study. This figure highlights the main results and conclusions drawn from the research.

## Highlights

- CD105^+^ fibroblasts induce breast tumor cells to migrate, proliferate, and express stemness and bone-like genes.
- Specific fibroblast subpopulations reshape tumor cells, promoting cancer progression.
- Understanding these interactions may help design therapies targeting tumor and supportive cells.

## 1 Introduction

Breast cancer progression is driven not only by tumor cell–intrinsic alterations but also by dynamic interactions with the stromal microenvironment, which critically regulate primary tumor growth, invasion, and metastatic dissemination (1–4). Among stromal components, fibroblasts are the predominant cell population, and most acquire an activated phenotype known as CAFs during tumor progression (5). CAFs actively shape tumor behavior through paracrine signaling, extracellular matrix remodeling, immune modulation, and metabolic crosstalk; however, they constitute a highly heterogeneous population whose functional diversity remains incompletely understood (6, 7).

Accumulating evidence indicates that CAF heterogeneity is influenced by tumor subtype, microenvironmental cues, and cellular origin, giving rise to stromal subpopulations with distinct—and sometimes opposing—biological functions (8–12). Despite this growing recognition, the identification of functionally relevant CAF subsets has been limited by the lack of markers beyond descriptive phenotyping. CD105 (endoglin), a co-receptor for transforming growth factor-β (TGF-β) and a canonical MSC marker, has emerged as a potential discriminator of CAF subpopulations with enhanced stem-like and pro-tumorigenic properties (13, 14). Consistent with this role, it regulates MSC proliferation, migration, differentiation, and stemness, and plays a key role in mediating stromal responses to TGF-β signaling within the tumor microenvironment (14, 15).

Previous work from our group demonstrated that early-stage breast tumor cells secrete chemotactic factors that recruit and activate stromal cells expressing CAF- and MSC-associated markers, including CD105 (16, 17). Importantly, elevated CD105 expression in intratumoral fibroblasts, not associated with the vasculature (CD34-negative) has been linked to developing metastasis—particularly bone metastasis—within a shorter timeframe and reduced survival, highlighting its potential as a prognostic marker and suggesting a role for CD105-positive CAFs in shaping tumor cell programs relevant to metastatic niche formation (18).

Despite these observations, it remains unclear whether CD105 expression functionally distinguishes CAF subpopulations. In particular, the differential contribution of CD105-positive versus CD105-negative fibroblasts to cancer cell behavior and the induction of osteogenic-related traits has not been directly examined in human primary breast tumors. Here, we aimed to characterize the phenotypic, molecular, and functional properties of CD105(+)/CD34 (–) and CD105 (–)/CD34(-) stromal cell subpopulations, and to determine how these fibroblast subpopulations differentially modulate breast cancer cell behavior, including the acquisition of osteoblast-like features relevant to metastatic progression.

## 2 Materials and methods

### 2.1 Sample selection

The inclusion criteria were samples from women with early clinical-pathological stage I/II invasive ductal breast carcinoma (IDC). The exclusion criteria included having received neoadjuvant therapy, the presence of another tumor, or an insufficient sample size (<1 cm). The samples were received consecutively, and only those that met the inclusion and exclusion criteria were used. Therefore, a prospective study was conducted using fibroblasts (mostly CAFs) obtained from primary tumor tissue extracted during surgery from 10 women aged 26 to 80 years with early clinical-pathological stage I/II invasive ductal breast carcinoma, luminal molecular subtype. Histological diagnosis of the tumor was made per the International Union Against Cancer classification system (19). The details of the clinical-pathological characteristics of the breast cancer patients (BCPs) are shown in **Table 1**.

**Table 1:** Details of clinicopathological characteristics of breast cancer patients included in the study.

Clinicopathological characteristics		Patients	
		N	%
Age (years)	<50	2	20%
	≥50	8	80%
Tumoral size (cm)	≤2	5	50%
	>2	5	50%
ER	Negative	0	0%
	Positive	10	100%
PR	Negative	0	0%
	Positive	10	100%
Her2/neu	Negative	9	90%
	Positive	1	10%
Histological grade	G1	0	0%
	G2	8	80%
	G3	2	20%
Regional lymph nodes	Negative	9	90%
	Positive	1	10%
Local relapse	Negative	10	100%
	Positive	0	0%
Metastatic recurrence	Negative	10	100%
	Positive	0	0%
Bone metastatic recurrence	Negative	10	100%
	Positive	0	0%
Visceral metastatic recurrence	Negative	10	100%
	Positive	0	0%
Breast cancer subtypes			
*Luminal A*		2	20%
*Luminal B*		8	80%
*Basal-like*		0	0%
*Her2/neu overexpression*		0	0%

ER, estrogen receptor, PR, progesterone receptor, Her2/neu, epidermal growth factor receptor 2.

Luminal breast cancer subtypes were identified as follows: i) Luminal A: This subtype is characterized by positive expression of estrogen receptor (ER) and/or progesterone receptor (PR), a low Ki-67 proliferation index (≤14%), and negative human epidermal growth factor receptor 2 (Her2) expression and ii) Luminal B: This subtype expresses ER and/or PR, but usually has a higher Ki-67 proliferation index (>14%) and positive/negative Her2 expression (+). The expression of ER, PR, and HER2/neu status was classified as negative or positive according to Wernicke M et al. (20) and the Ki-67 proliferation was determined by an immunoperoxidase procedure using a monoclonal antibody, Ki-67, which reacts with a nuclear antigen in proliferating cells, according to S. Crispino et al. (21). Tumor samples were provided by the Pathology Service of the Italian Hospital, CABA. After surgery, all patients underwent personalized treatment, which involved a combination of hormonal therapy, radiotherapy, and/or chemotherapy and immunotherapy. The treatment plan was tailored based on individual clinical and histopathological characteristics, following the guidelines recommended by the European Society for Medical Oncology (22, 23). Ethical approval for this study was obtained from the Hospital Italiano (Hospital Italiano approval: no5009). Informed consent was obtained from all individual participants included in the study and the research adhered to the principles outlined in the Helsinki Declaration. To safeguard patient privacy, medical records were anonymized using a numerical code.

### 2.2 Isolation and expansion of fibroblasts from primary breast tumor tissue

Immediately after surgery, breast tumor specimens were processed under sterile conditions and enzymatically digested with collagenase/hyaluronidase (StemCell Technologies) overnight at 37°C. Stromal cells were enriched by sequential low-speed centrifugation steps and resuspended in supplemented α-MEM. Cell number and viability were assessed by trypan blue exclusion. Primary cultures were established in α-MEM supplemented with antibiotics/antimycotics and 20% fetal bovine serum (FBS), maintained at 37°C and 5% CO_2_, and medium was refreshed weekly. Adherent fibroblast-like cells were expanded by routine passaging at 70–80% confluence; cells from the second subculture were subsequently used for the isolation of CD105(+)/CD34(−) and CD105(−)/CD34(−) fibroblast subpopulations. Detailed processing and centrifugation steps are provided in the **Supplementary Materials**.

### 2.3 Separation of CD105(+)/CD34 (–) and CD105(-)/CD34(-) fibroblasts

CD105(+)/CD34(−) and CD105(−)/CD34(−) fibroblast subpopulations were isolated from second-passage CAFs using magnetic-activated cell sorting (MACS) based on sequential CD34 and CD105 selection. Briefly, CD34-negative stromal cells were first enriched and subsequently separated into CD105-positive and CD105-negative fractions. Both subpopulations were expanded under standard culture conditions in supplemented α-MEM containing 20% FBS and passaged upon reaching 70–80% confluence. To enrich for fibroblasts derived from MSCs, cells were transiently cultured at low density, followed by further expansion. Fifth-passage cultures were used for all subsequent assays. CM were collected from the final 48 h of serum-free culture at 70–80% confluence and pooled for proteomic analyses and functional assays on breast cancer cell lines. Detailed separation and expansion procedures are provided in the **Supplementary Materials**.

### 2.4 Analysis of CD105 and CD34 expression in cancer-associated fibroblasts from paraffin-embedded breast cancer samples

CD105 and CD34 expression in CAFs was analyzed by double immunohistochemistry on paraffin-embedded breast cancer samples. Appropriate negative controls were included, and all analyses were performed in duplicate for each sample. Detailed information on antibodies and detection systems is provided in the **Supplementary Materials**.

### 2.5 Cell culture protocol for breast cancer cell lines

Human breast cancer cell lines MCF-7 and MDA-MB-231 (ATCC) were cultured under standard conditions in supplemented DMEM/F12 containing 10% FBS at 37°C in a humidified 5% CO_2_ atmosphere. MCF-7 cultures were additionally supplemented with insulin. Cells were routinely passaged at confluence, and viability was assessed by trypan blue exclusion. For functional and gene expression assays, cells were used up to the fourth passage. Detailed culture conditions are provided in the **Supplementary Materials**.

### 2.6 Study of the phenotype of CD105(+)/CD34(-) and CD105(-)/CD34(-) fibroblastic subpopulations

The immunophenotype of CD105(+)/CD34(−) and CD105(−)/CD34(−) subpopulation fractions was analyzed by flow cytometry using a panel of antibodies targeting classical MSC markers and activated CAF markers. Cells were stained under standard conditions, including appropriate isotype controls, and both surface and intracellular markers were evaluated. Data were acquired on a FACSCanto II flow cytometer and analyzed with FlowJo software. Relative fluorescence indices were calculated using isotype controls. Experiments were performed in duplicate using fibroblast preparations obtained from breast cancer samples from 10 patients. Detailed antibody information and staining procedures are provided in the **Supplementary Materials**.

### 2.7 Study of self-renewal, CFU-F assay and morphology of stromal cells within colonies

The self-renewal capacity of CD105(+)/CD34(−) and CD105(−)/CD34(−) fibroblast subpopulations was assessed using the colony-forming unit–fibroblast (CFU-F) assay. Cells were seeded at low density and cultured under standard conditions, and colonies were evaluated after 14 days. Colonies containing ≥50 cells were scored as CFU-Fs, and colony-forming efficiency was calculated. In addition, stromal cell density and morphological features of fibroblast-like cells within CFU-F colonies were quantified using digital image analysis. Assays were performed in duplicate using fibroblast preparations obtained from breast cancer samples from 10 patients. Detailed experimental procedures are provided in the **Supplementary Materials**.

### 2.8 Assessment of cell viability as an indirect measure of proliferation capacity

Cell proliferation of CD105(+)/CD34(−) and CD105(−)/CD34(−) fibroblast subpopulations was evaluated using a colorimetric MTS-based assay. Cells were seeded in 96-well plates, synchronized under serum-free conditions, and subsequently stimulated with serum-containing medium. Basal controls were maintained under serum-free conditions. Cell viability, used as an indirect measure of proliferation, was quantified by measuring absorbance at 490 nm. Experiments were performed in triplicate. Detailed experimental conditions are provided in the **Supplementary Materials**.

### 2.9 Study of cell cycle

Cell cycle distribution of CD105(+)/CD34(−) and CD105(−)/CD34(−) fibroblast subpopulations was analyzed by propidium iodide–based flow cytometry following standard protocols. Samples were acquired using a FACSCanto II flow cytometer, and data were analyzed with FlowJo software. Experiments were performed twice for each sample. Detailed staining and acquisition procedures are provided in the **Supplementary Materials**.

### 2.10 Gene expression study

#### 2.10.1 RNA extraction and cDNA synthesis

Total RNA was extracted from CD105(+)/CD34(−) and CD105(−)/CD34(−) fibroblast subpopulations at the fifth passage using a commercial reagent, and cDNA was synthesized from 1 µg of RNA using a reverse transcription kit according to the manufacturer’s instructions. Detailed extraction and reverse transcription conditions are provided in the **Supplementary Materials**.

#### 2.10.2 Quantitative real-time PCR

Gene expression analysis was performed by quantitative real-time PCR using SYBR Green chemistry under standard amplification conditions, followed by melting curve analysis. Cycle threshold values were normalized to the GAPDH reference gene. The expression of genes related to stemness, osteogenic differentiation, osteoclastogenesis regulation, migration, and tumor progression was evaluated. Primer sequences are provided in **Supplementary Table 1**. Experiments were conducted in duplicate for each sample.

### 2.11 Study of oxidative stress [mitochondrial ROS (superoxide anion) and total ROS]

Intracellular oxidative stress was assessed in CD105(+)/CD34(−) and CD105(−)/CD34(−) fibroblast subpopulations by flow cytometry using fluorescent probes for total and mitochondrial reactive oxygen species (ROS). Total ROS and mitochondrial superoxide levels were detected using CellROX and MitoSOX dyes, respectively. Fluorescence intensity was quantified in viable (4′,6-diamidino-2-phenylindole [DAPI]-negative) cells using a FACSCanto II flow cytometer, and data were analyzed with FlowJo software. Experiments were performed twice for each sample. Detailed staining and acquisition procedures are provided in the **Supplementary Materials**.

### 2.12 Study of cellular senescence

Cellular senescence in CD105(+)/CD34(−) and CD105(−)/CD34(−) fibroblast subpopulations was evaluated using senescence-associated β-galactosidase (SA-β-gal) staining. Cells were cultured under standard conditions, fixed, and stained following established protocols. SA-β-gal–positive cells were visualized by light microscopy. Experiments were performed twice for each sample. Detailed staining procedures are provided in the **Supplementary Materials**.

### 2.13 Secretome study

For secretome analysis, CM collected after 48 h of serum-free culture from CD105(+)/CD34(−) and CD105(−)/CD34(−) fibroblast subpopulations derived from 10 patients were pooled and concentrated. Proteomic profiling was performed using a label-free quantification (LFQ) approach based on high-resolution mass spectrometry. Protein identification and relative quantification were conducted using standard bioinformatic pipelines, and differentially expressed proteins between both subpopulations were identified. Functional interaction networks were constructed using STRING software. Detailed sample preparation, electrophoresis, and mass spectrometry procedures are provided in the **Supplementary Materials**.

### 2.14 Effect of CM pool from CD105(+)/CD34(-) and CD105(-)/CD34(-) fibroblastic subpopulations on breast cancer cells of the MCF-7 and MDA-MB231 cell lines

#### 2.14.1 Migration of breast cancer cells

Cell migration was evaluated using a wound healing assay, in which wound closure was used as an indicator of cellular motility. Confluent monolayers of MCF-7 and MDA-MB-231 breast cancer cells were mechanically scratched, and the culture medium was replaced to remove cellular debris. Pooled CM from CD105(+)/CD34(−) and CD105(−)/CD34(−) fibroblast subpopulations (n = 10) were applied to the wounded monolayers. Supplemented α-MEM containing 10% FBS and supplemented α-MEM alone were used as positive and basal controls, respectively. Cell migration was quantified by measuring wound closure between time 0 (T0) and 12 h (T12) using ImageJ software. Each assay was performed in duplicate and repeated seven times.

#### 2.14.2 Assessment of cell viability as an indirect measure of proliferation capacity of breast cancer cells

Breast cancer cell proliferation was assessed using the same assay applied to fibroblasts. MCF-7 and MDA-MB-231 cells were seeded, serum-starved, and subsequently treated with pooled CM from CD105(+)/CD34(−) or CD105(−)/CD34(−) fibroblasts, or with appropriate control media. Cell viability was used as an indirect measure of proliferation. All experiments were performed in triplicate and repeated four times. Detailed experimental procedures are provided in the **Supplementary Materials**.

#### 2.14.3 Gene expression in breast cancer cells

Gene expression analysis in breast cancer cells was performed by quantitative real-time PCR following treatment with pooled CM from CD105(+)/CD34(−) or CD105(−)/CD34(−) fibroblasts, or with basal medium alone. MCF-7 and MDA-MB-231 cells were exposed to the respective treatments for 48 h prior to RNA extraction. The expression of genes associated with stemness and osteogenic differentiation was evaluated (see **Supplementary Table 1**). Experiments were conducted in duplicate and repeated seven times. Detailed experimental procedures are provided in the **Supplementary Materials**.

### 2.15 Statistical analysis

The analysis was consistently conducted between two cell types: CD105(+)/CD34(-) stromal cells and CD105(-)/CD34(-) stromal cells. The results were expressed as the mean ± standard error (SEM) when appropriate. The Shapiro-Wilk test for normality was used to determine if data were normally distributed and then analyzed with the corresponding test. For parametric data, differences between groups were assessed using an unpaired t-test, with Welch’s correction when necessary. Two-way ANOVA was conducted for multiple comparisons, followed by *post hoc* multiple comparisons using the Bonferroni method.

Statistical analyses were performed using GraphPad Prism 6 software (version 6.01, GraphPad Software, La Jolla CA, USA). Statistically significant differences were considered when p < 0.0500.

## 3 Results

### 3.1 Phenotypic characterization of CD105(+)/CD34(-) and CD105(-)/CD34(-) fibroblastic subpopulations

#### 3.1.1 Before column separation of fibroblast subpopulations

The percentage of CD105(+)/CD34(−) fibroblasts was first evaluated in fresh breast tumor tissue samples prior to magnetic column separation. Flow cytometry analysis showed that 28.80 ± 4.52% of fibroblasts expressed CD105, whereas 4.14 ± 1.03% expressed CD34 (X ± SE, n = 10). Immunohistochemical analysis performed on paraffin-embedded tumor sections from the same patients revealed that 19.70 ± 3.95% of stromal fibroblasts were CD105-positive and CD34-negative, excluding vascular-associated cells. These analyses confirmed the presence of a measurable CD105(+)/CD34(−) fibroblast population within the breast tumor stroma prior to cell isolation.

#### 3.1.2 After isolation of both subpopulations [CD105(+)/CD34(-) and CD105(-)/CD34(-)]

**Figure 1:**
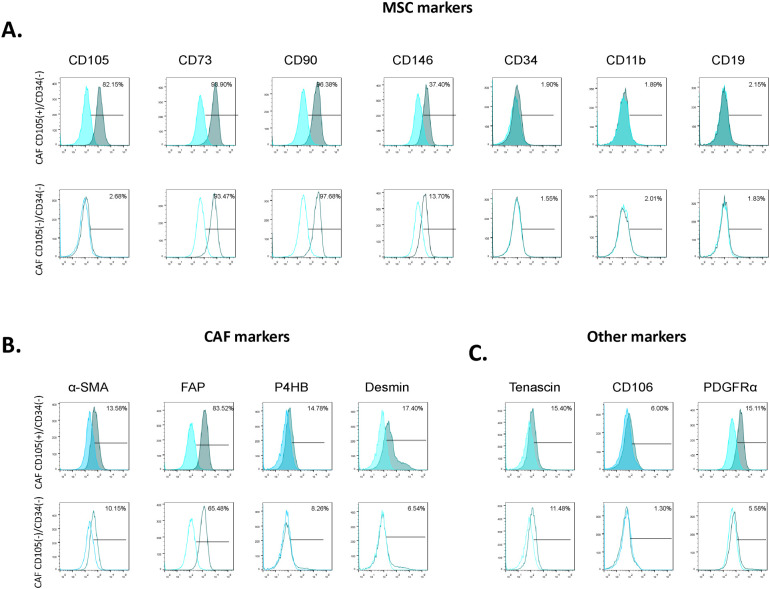
Phenotypic characterization of CD105(+)/CD34(-) and CD105(-)/CD34(-) fibroblastic subpopulations. **(A)** Classical MSC markers. **(B)** CAF markers. **(C)** Markers associated with fibroblast activation and tumor-related functions. Representative flow cytometry histograms depicting the surface antigens of both subpopulations, obtained from a representative patient with breast cancer (BCPs) (n=10). (□) Isotype control.

Following magnetic separation, both fibroblast subpopulations were analyzed by flow cytometry. High percentages of CD73 and CD90-positive cells were detected in both CD105(+)/CD34(−) and CD105(−)/CD34(−) fibroblasts, with no significant differences between groups (**Figure 1**, **Table 2**). A significantly higher percentage of CD146-positive cells was detected in the CD105(+)/CD34(−) fibroblast subpopulation compared with CD105(−)/CD34(−) fibroblasts (**Figure 1**, **Table 2**). The expression of negative MSC markers (CD34, CD11b, and CD19) was low in both subpopulations, with no significant differences observed. Both fibroblast subpopulations expressed CAF-associated markers alpha-Smooth Muscle Actin (α-SMA) and fibroblast activation protein (FAP), with comparable percentages of positive cells (**Figure 1**, **Table 2**). In both groups, the proportion of FAP-positive cells was higher than that of α-SMA-positive cells. Analysis of additional markers associated with fibroblast activation and tumor-related functions showed higher percentages of CD106-, platelet-derived growth factor receptor alpha (PDGFRα)-, desmin-, and prolyl 4-hydroxylase subunit beta (P4HB)-positive cells in the CD105(+)/CD34(−) fibroblasts compared with the CD105(−)/CD34(−) subpopulation (**Figure 1**, **Table 2**). No significant differences were observed in relative fluorescence intensity (RFI) for any marker between the two subpopulations (**Table 2**).

**Table 2:** Phenotypic analysis.

Markers	CD105(+)/CD34(-)	CD105(-)/CD34(-)	p	CD105(+)/CD34(-)	CD105(-)/CD34(-)	p
	% positive cells	% positive cells		RFI	RFI	
CD105	81.52 ± 0.86	5.93 ± 1.36	<0.0001	1.57 ± 0.30	1.33 ± 0.27	0.5749
CD73	94.24 ± 1.41	93.47 ± 1.18	0.8187	3.25 ± 0.70	3.01 ± 0.77	0.8284
CD90	95.88 ± 1.37	97.68 ± 0.23	0.3903	2.86 ± 0.43	3.16 ± 0.82	0.7593
CD146	13.65 ± 2.69	4.25 ± 1.42	0.0164	1.27 ± 0.47	1.25 ± 0.24	0.9854
CD34	4.06 ± 1.03	3.51 ± 1.06	0.3975	1.04 ± 0.09	0.95 ± 0.04	0.3896
CD11b	3.78 ± 1.34	3.41 ± 1.19	0.5180	0.69 ± 0.31	0.93 ± 0.04	0.5922
CD19	2.52 ± 0.08	2.37 ± 0.05	0.1240	1.26 ± 0.58	0.93 ± 0.11	0.6154
α-SMA	14.36 ± 2.74	10.93 ± 2.81	0.3989	3.54 ± 1.54	4.50 ± 1.40	0.6998
FAP	80.37 ± 4.29	64.80 ± 8.39	0.1334	1.95 ± 0.37	2.76 ± 1.00	0.4963
P4HB	15.56 ± 1.59	7.89 ± 1.95	0.0281	2.57 ± 1.07	3.50 ± 1.03	0.6788
DESMIN	18.22 ± 1.42	7.67 ± 2.35	0.0040	0.68 ± 0.27	1.09 ± 0.05	0.2713
TENASCIN	27.91 ± 7.46	16.94 ± 5.86	0.3809	1.51 ± 0.74	0.83 ± 0.21	0.4609
CD106	5.78 ± 0.35	1.88 ± 0.17	0.0010	1.46 ± 0.84	0.57 ± 0.07	0.3678
PDGFRα	16.05 ± 2.04	5.96 ± 1.39	0.0040	0.94 ± 0.05	0.87 ± 0.10	0.5956

Percentage (%) and relative fluorescence index (RFI) of CD105(+)/CD34(-) and CD105(-)/CD34(-) fibroblastic subpopulations of early breast cancer patients (BCPs) (n=10).

### 3.2 Self-Renewal, CFU-F assay of CD105(+)/CD34(-) and CD105(-)/CD34(-) fibroblastic subpopulations and morphology of stromal cells within colonies

**Figure 2:**
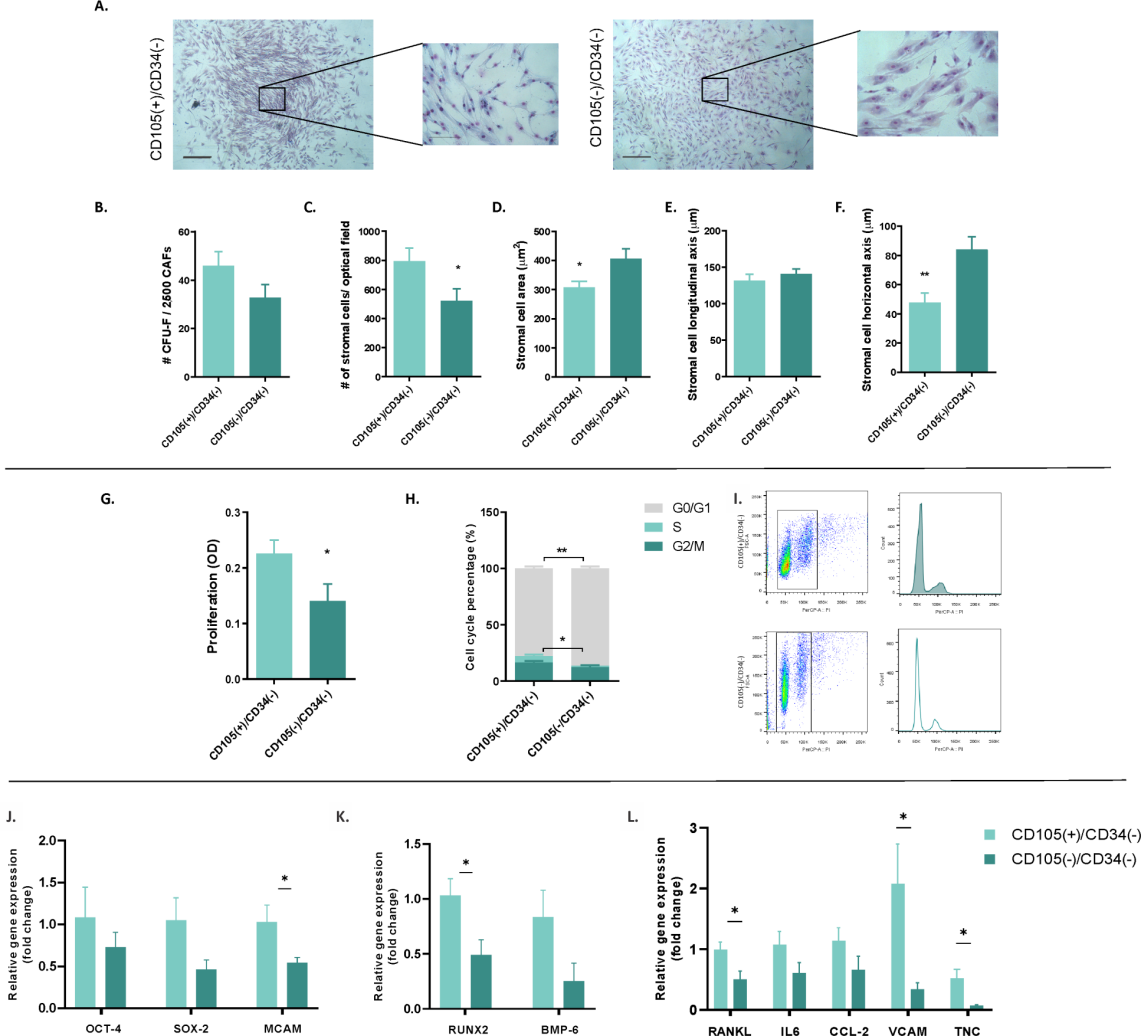
Self-renewal, proliferation, cell cycle, and expression of stemness, osteogenic, osteoclastogenic, and migratory factors in early breast cancer patients (BCPs). **(A)** Self-renewal, assessed by colony-forming unit–fibroblastic (CFU-F) formation, of CAF CD105(+)/CD34(–) (left panel) and CD105(–)/CD34(–) (right panel) fibroblastic subpopulations (n=10). Photographs showing the size of each CFU-F and the stromal cell shapes observed in a representative BCP. Giemsa staining (40x and 200X in the inset). The scale bar represents 60 μm, and 200 μm in the inset. **(B)** Number (#) of observed CFU-F (p= 0.1133). **(C)** Number (#) of CD105(+)/CD34(-) and CD105(-)/CD34(-) fibroblasts per microscope field in each CFU-F (*p=0.0412). **(D)** Area of stromal cells in typical regions of each CFU-F culture from BCPs (*p= 0.0272). **(E)** Longitudinal/major elliptical axis of stromal cells in typical regions of CFU-F cultures from BCPs (p=0.4976). **(F)** Horizontal/minor elliptical axis of stromal cells in typical regions of BCPs CFU-F culture from BCPs (**p=0.0049). All values are expressed as mean ± SEM. Statistical analysis: unpaired t-test with Welch correction. Asterisks indicate significant differences. **(G)** Proliferation of CD105(+)/CD34(-) and CD105(-)/CD34(-) fibroblasts (n=10). Values are expressed as mean ± SEM. Statistical analysis: unpaired t-test with Welch correction (*p=0.0460). O.D: optical density. **(H)** Cell cycle analysis. Histogram representing the percentage of CD105(+)/CD34(-) and CD105(-)/CD34(-) fibroblasts in each phase of the cell cycle (G0/G1, S, and G2/M) (n=10). Values are expressed as mean ± SEM. Statistical analysis: unpaired t-test with Welch correction. Asterisks indicate significant differences (*p=0.0207 and **p=0.0067). **(I)** Gating strategy to identify CAFs in different cell cycle phases by FACS, shown for one representative BCP. **(J)** Gene expression of stemness (self-renewal and pluripotency) factors such as OCT-4, SOX-2, and MCAM (CD146) using quantitative real-time polymerase chain reaction (q-PCR) (n=10). **(K)** Gene expression of osteogenic factors, such as RUNX-2 and BMP6, as well as osteoclastogenic factors such as CCL-2 and IL-6 (n=10). **(L)** Gene expression of factors related to the migration process of mesenchymal stromal cells as well as breast cancer cells, and tumor evolution, such as VCAM (CD106), RANKL, CCL-2, IL-6, and TNC (n=10). All results were normalized to the reference gene GAPDH. Values are expressed as Mean ± SEM. Statistical analysis: unpaired Student’s t-test with Welch correction. Asterisks indicate significant differences (*p < 0.0500).

Both fibroblast subpopulations generated a comparable number of CFU-F per 2,500 plated cells (**Figures 2A, B**). Quantitative analysis revealed a significantly higher number of stromal cells per optical field within CFU-F derived from CD105(+)/CD34(−) fibroblasts compared with CD105(−)/CD34(−) fibroblasts (p = 0.0412) (**Figure 2C**). Morphometric analysis showed that cells within CD105(+)/CD34(−) CFU-F exhibited a smaller cell area (p = 0.0272), mainly due to a reduced horizontal axis length (p = 0.0049) (**Figures 2D–F**).

### 3.3 Cell viability as an indirect measure of proliferation capacity and cell cycle in CD105(+)/CD34(-) and CD105(-)/CD34(-) fibroblast subpopulations

CD105(+)/CD34(-) fibroblasts exhibited a higher cell viability rate compared to the CD105(-)/CD34(-) population (p=0.0460) (**Figure 2G**). Cell cycle analysis revealed that CD105(+)/CD34(-) fibroblasts also showed a higher percentage of cells in the S phase compared to CD105(-)/CD34(-), and a lower proportion of cells in the G0/G1 phase (p=0.0207 and p=0.0067; respectively) (**Figure 2H, I**).

### 3.4 Expression of stemness genes (self-renewal and multipotentiality, particularly osteoblastic differentiation), as well as genes related to osteoclastogenesis regulation, migration capacity, and tumor evolution in CD105(+)/CD34(-) and CD105(-)/CD34(-) fibroblastic subpopulations

Gene expression analysis showed higher expression levels of melanoma cell adhesion molecule (MCAM) [CD146], runt-related transcription factor 2 (RUNX2), receptor activator of nuclear factor-kappa β ligand (RANKL), vascular cell adhesion molecule (VCAM) [CD106], and tenascin C (TNC) in CD105(+)/CD34(−) compared to CD105(-)/CD34(-) fibroblasts (p=0.049, p=0.0237, p=0.0293, p=0.0303, and p=0.0251, respectively) (**Figures 2J–L**). Expression of the other genes like sry-box transcription factor 2 (SOX2), octamer-binding transcription factor 4 (OCT4), bone morphogenetic protein (BMP)6, chemokine (C-C motif) Ligand 2 (CCL-2), and interleukin 6 [IL-6]) was present but did not show significant differences between the two fibroblast subpopulations.

### 3.5 Oxidative stress study in CD105(+)/CD34(-) and CD105(-)/CD34(-) fibroblastic subpopulations

**Figure 3:**
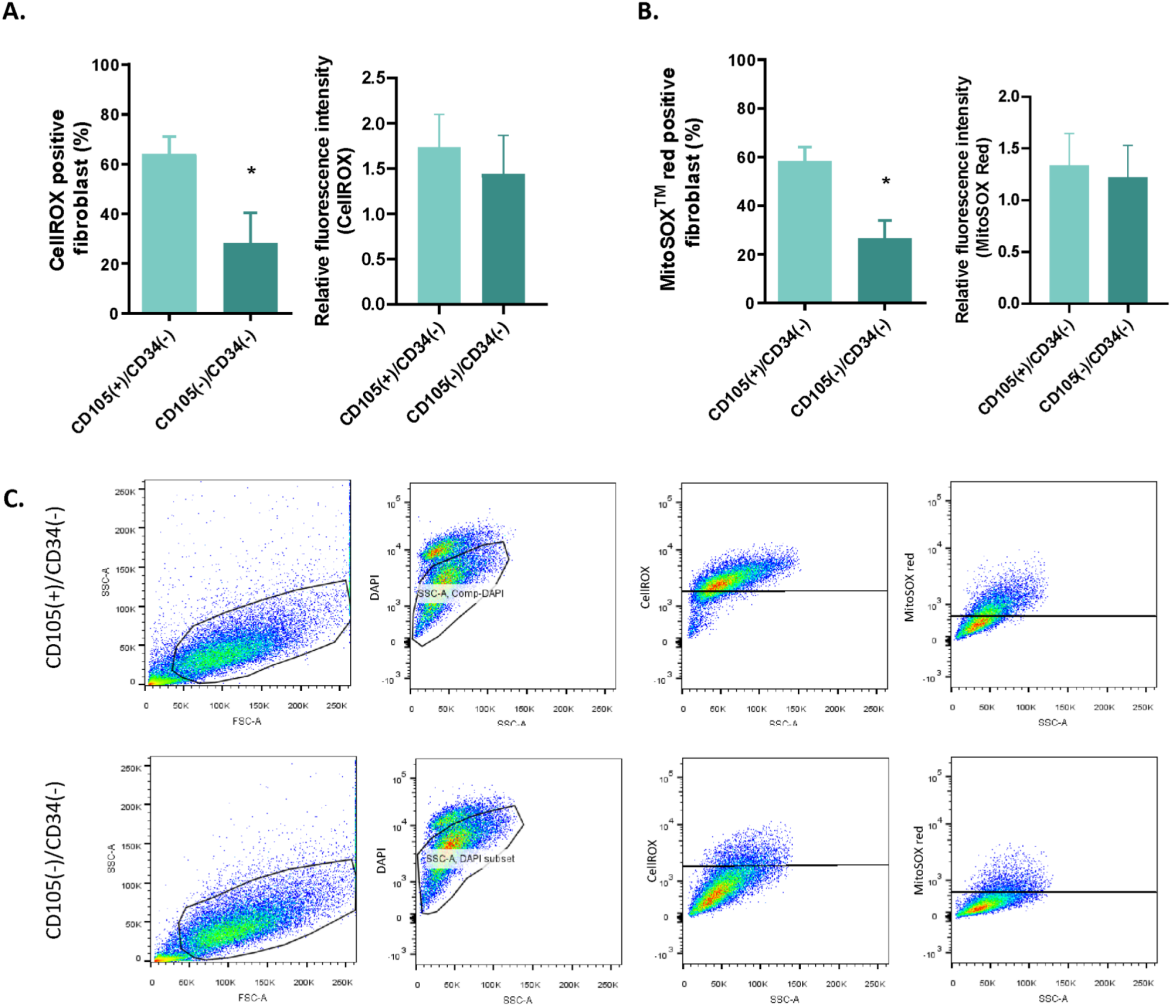
Cellular reactive oxygen species (ROS) levels in CD105(+)/CD34(-) and CD105(-)/CD34(-) fibroblastic subpopulation isolated from the primary tumor of breast cancer patients (BCPs). **(A)** Percentage of cells positive for total ROS. Staining with the CellROX Kit for CD105(+)/CD34 (-) and CD105(-)/CD34(-) fibroblasts from BCPs (n=10). **(B)** Percentage of cells positive for mitochondrial ROS. Staining with the MitoSox Red Kit for CD105(+)/CD34(-) and CD105(-)/CD34(-) fibroblasts from BCPs (n=10). Values are expressed as Mean ± SEM. Statistical analysis: unpaired Student’s t-test with Welch correction. Asterisks indicate significant differences (*p=0.0393 and *p=0.0110; respectively). **(C)** Gating strategy to identify total ROS (CellROX) and mitochondrial ROS (MitoSOX Red) in CAF subpopulations in breast cancer by FACS, shown for one representative BCP. Cells were gated on DAPI to exclude dead cells.

CD105(+)/CD34(-) CAFs showed a significantly higher percentage of cells positive for both CellROX and MitoSOX probes (p=0.0393 and p=0.0110, respectively) compared to the values of CD105(-)/CD34(-) (**Figures 3A–C**). Additionally, we found similar values of mean fluorescent intensity (level of ROS per cell) for both probes regardless of the type of fibroblastic subpopulations (**Figures 3A–C**).

### 3.6 Cellular senescence through SA-β-galactosidase detection in CD105(+)/CD34(-) and CD105(-)/CD34(-) fibroblastic subpopulations

Both CD105(+)/CD34(-) and CD105(-)/CD34(-) fibroblasts showed a comparable percentage of cells positive for SA-β-galactosidase, indicating a similar level of cellular senescence (**Supplementary Figure 1**).

### 3.7 Analysis of the secretoma in the CM of CD105(+)/CD34(-) and CD105(-)/CD34(-) fibroblastic subpopulations

**Figure 4:**
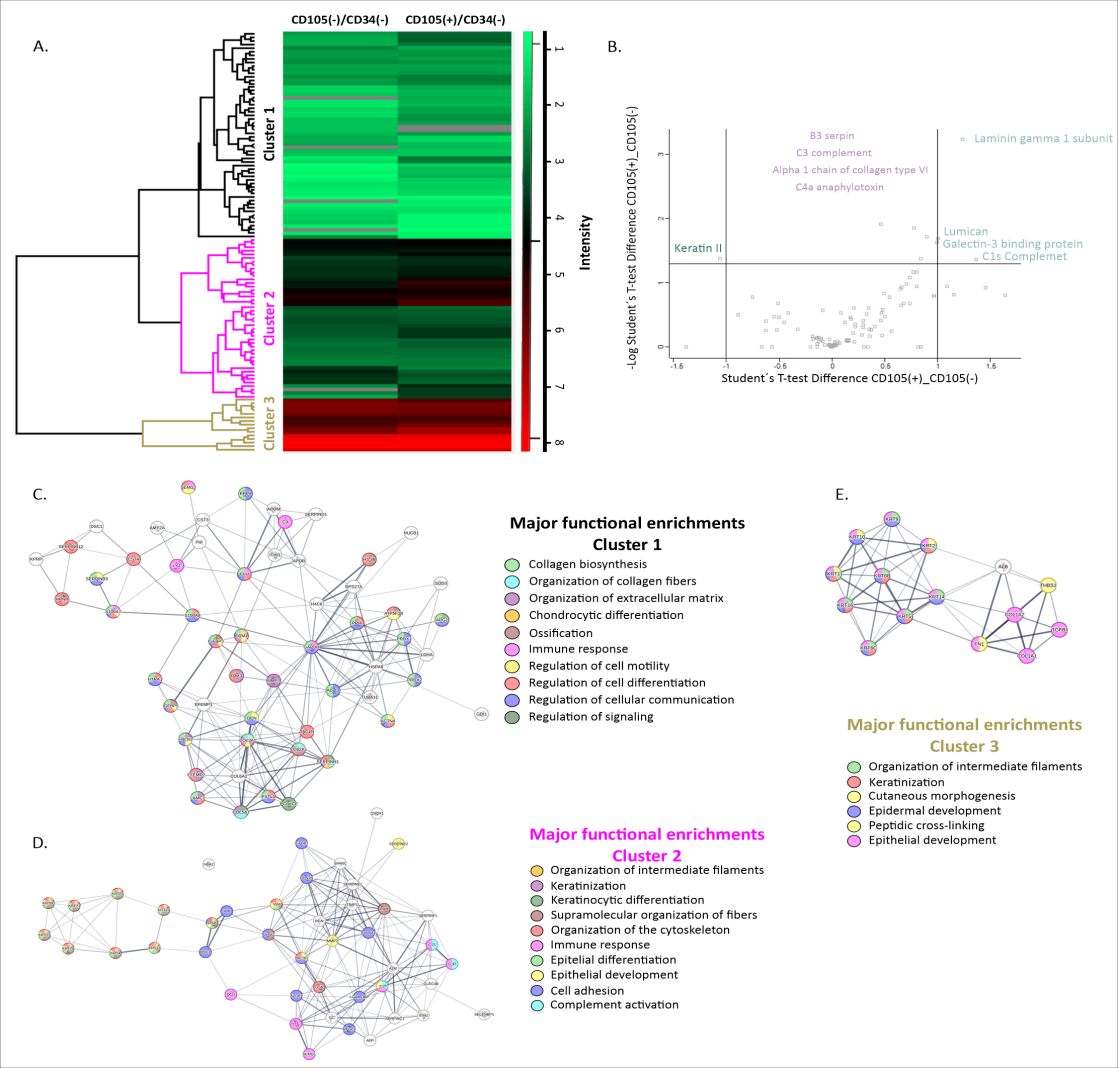
Secretome of CD105(+)/CD34(-) and CD105(-)/CD34(-) fibroblastic subpopulations in breast cancer patients. **(A)** Heatmap showing the proteins included in the first (Cluster 1), second (Cluster 2), and third (Cluster 3) clusters displaying differential abundance between CD105⁺/CD34⁻ and CD105⁻/CD34⁻ fibroblast subpopulations. In each heatmap, high and low abundance levels are depicted in red and green, respectively (scale to the right of the heatmap). **(B)** Representation (scatter plot) of the proteins found in the secretome of CD105(+)/CD34(-) and CD105(-)/CD34(-) fibroblastic subpopulations (pool of 10 CM). Proteins located in the upper right and left quadrants are those with a relative change greater than 2 (in the plot, less than -1 or greater than 1, logarithmic scale) and a p-value < 0.0500 (in the plot, greater than 1.3, logarithmic scale). In the upper right quadrant are proteins increased in the CD105(+)/CD34(-) fibroblast fraction, in the upper left quadrant are proteins increased in CD105(-)/CD34(-) fibroblasts, while in the upper middle quadrant are proteins found abundant but with no difference in abundance between both fibroblastic subpopulations. Statistical test: Student’s t-test. **(C)** Interaction network among the proteins found in the first cluster. The interaction network was generated using the String online tool, considering both functional and physical associations of proteins. The thickness of the line connecting each protein indicates the strength of data support (confidence). The main biological processes (gene ontology) are detailed with colored circles. **(D)** Interaction network among the proteins found in the second cluster. **(E)** Interaction network among the proteins found in the third cluster.

Proteomic analysis of the CM from CD105(+)/CD34(−) and CD105(−)/CD34(−) fibroblast subpopulations identified three major protein clusters common to both secretomes (**Figure 4A**). The first cluster comprised proteins involved in extracellular matrix components and cytoskeletal-associated proteins, including S100A7, tubulin-1, serpin B3, insulin-like growth factor-binding protein (IGFBP)-3, IGFBP5, collagen type III alpha 1 (COL3A1), and decorin. Proteins related to immune-associated pathways were also detected within this cluster, such as lysozyme C, S100A7, complement component C9, and clusterin (**Figures 4A–C**). The second cluster included proteins associated with intermediate filament organization, keratinization, and cytoskeleton organization, as well as proteins involved in epithelial-related processes and complement activation. Among these were secreted protein acidic and rich in cysteine (SPARC), galectin-3–binding protein (Gal-3BP), and lumican (**Figure 4D**). The third cluster was mainly composed of proteins associated with keratinization and epidermal development, which represented the most abundant proteins detected in the secretome (**Figure 4E**). Comparative analysis of protein abundance revealed distinct expression patterns between the two fibroblast subpopulations. Statistically significant differences were observed for laminin, lumican (gamma 1 subunit), Gal-3BP, and complement component C1s in the CD105(+)/CD34(−) fibroblasts, whereas keratin II was significantly enriched in the CD105(−)/CD34(−) fibroblasts (**Figure 4B**).

### 3.8 Effect of CM pool from CD105(+)/CD34(-) and CD105(-)/CD34(-) fibroblastic subpopulations on human breast cancer cells of the MCF-7 and MDA-MB231 cell lines

#### 3.8.1 Migration study

**Figure 5:**
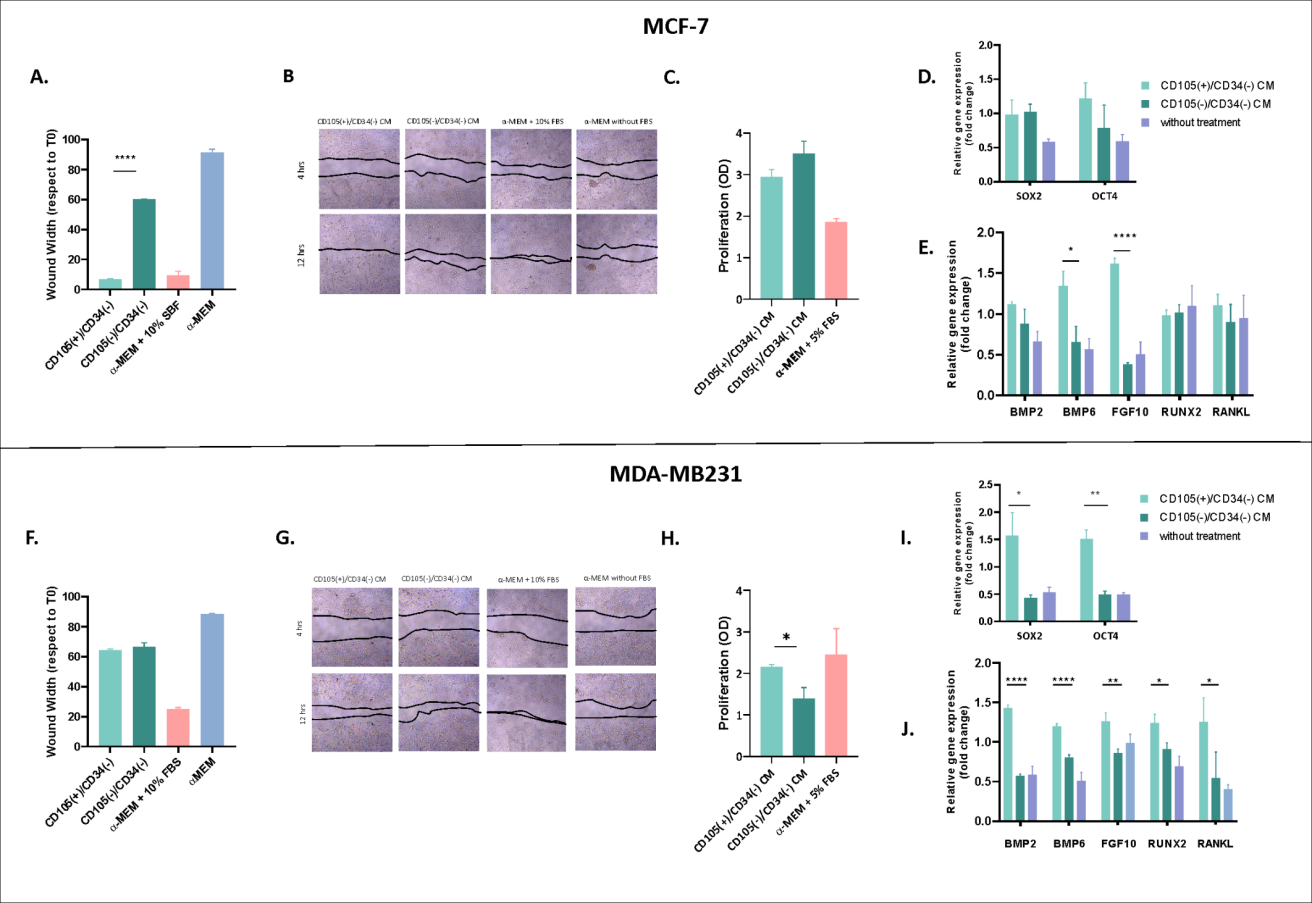
Impact of conditioned media (CM pool derived from CD105(+)/CD34(-) and CD105(-)/CD34(-) fibroblastic subpopulations of early breast cancer (BCPs) patients on the migration, proliferation, and gene expression of human breast cancer cells from MCF-7 and MDA-MB231 lines. **(A, F)** Study of migration through wound healing assay. Graph representing the wound width measured at the final time point (12 hrs) (normalized to time 0) under different conditions [CM CD105(+)/CD34(-), CM CD105(-)/CD34(-), positive control (α-MEM 10% fetal bovine serum (FBS), and negative control (α-MEM only)] (n=7). The values are expressed as mean ± SEM. A statistical analysis was conducted using two-way ANOVA, followed by *post hoc* multiple comparisons using the Bonferroni method. Asterisks indicate significant differences (****p < 0.0001). Representative images of the wound area at time 1 (4 hrs) and the final time point (12 hrs) under each condition. MCF-7 is shown in the top panel **(A, B)** and MDA-MB231 is shown in the down panel **(F, G)**. **(C, H)** Study the proliferation. Treatment under different conditions during 48 hours: CM CD105(+)/CD34(-), CM CD105(-)/CD34(-), α-MEM only (basal or negative control) and α-MEM + 5% FBS (positive control) (n=4). The values are expressed as mean ± SEM. A statistical analysis was conducted using two-way ANOVA, followed by *post hoc* multiple comparisons using the Bonferroni method. Asterisks indicate significant differences (*p = 0.0433). **(D, E)** Study of gene expression. Treatment under different conditions during 48 hs: CM CD105(+)/CD34(-), CM CD105(-)/CD34(-), α-MEM only (basal or negative control) and α-MEM + 10% FBS (positive control). The expression of stemness (self-renewal and pluripotency) factors, including SOX-2 and OCT-4, as well as the expression of osteo-chondrogenic differentiation and bone mineralization factors such as BMP2, BMP6, FGF10, RUNX2, and RANKL, was assessed in MCF-7 **(D, E)** and MDA-MB231 **(I, J)** tumor cell lines using quantitative real-time polymerase chain reaction (q-PCR) (n=7). All results were normalized to the reference gene GAPDH. Values are expressed as Mean ± SEM. Statistical analysis: unpaired Student’s t-test with Welch correction. Asterisks indicate significant differences (*p < 0.0500, ** p < 0.0100, ****p <0.0001). The CM pools of the CD105(+)/CD34(-) and CD105(-)/CD34(-) fibroblastic subpopulations were obtained from 10 primary breast tumors of early BCPs.

CM derived from CD105(+)/CD34(−) fibroblasts induced a higher degree of wound closure in MCF-7 cells compared to CM from CD105(−)/CD34(−) fibroblasts (**Figures 5A, B**). In MDA-MB-231 cells, no significant differences were observed between the effects of CM from either fibroblast subpopulation (**Figures 5F, G**). In this cell line, wound closure in response to both CM conditions was lower than that observed with the 10% FBS positive control.

#### 3.8.2 Viability study as an indirect measure of proliferation capacity

CM from CD105(+)/CD34(−) fibroblasts induced higher viability in MDA-MB-231 cells compared with CM from CD105(−)/CD34(−) fibroblasts (p = 0.0433) (**Figure 5H**). No significant differences were observed in MCF-7 cells, although both CM increased viability relative to controls (**Figure 5C**).

#### 3.8.3 Gene expression study

In MDA-MB-231 cells, CM from CD105(+)/CD34(−) fibroblasts increased SOX2 and OCT4 expression compared with CM from CD105(−)/CD34(−) fibroblasts (**Figure 5I**). No differences were observed in MCF-7 cells (**Figure 5D**). CM from CD105(+)/CD34(−) fibroblasts increased BMP6 and fibroblast growth factor (FGF)10 expression in MCF-7 cells (p = 0.0196 and p < 0.0001, respectively) (**Figure 5E**). In MDA-MB231 cells, CM from CD105(+)/CD34(−) fibroblasts increased expression of BMP2, BMP6, FGF10, RUNX2, and RANKL was observed (**Figure 5J**).

## 4 Discussion

Breast cancer progression is critically shaped by the tumor microenvironment, where CAFs emerge as key regulators of tumor growth, stromal remodeling, and metastatic dissemination (24). Given their significant heterogeneity, not all fibroblast subpopulations across breast cancer subtypes are fully understood, particularly those potentially derived from bone marrow-MSCs, which represent a major source of stromal cells within the tumor microenvironment (9, 25–28).

In the present study, we identified two fibroblast subpopulations within the breast tumor stroma—CD105(+)/CD34(–) and CD105(–)/CD34(–)—that share several mesenchymal features but differ in key phenotypic and functional traits. While both subpopulations expressed CD73 and CD90 and lacked hematopoietic markers, only CD105(+)/CD34(–) fibroblasts expressed the canonical MSC marker profile (29). This phenotype is consistent with previous reports describing mesenchymal progenitor-like cells isolated from breast tumors that retain stem cell characteristics and fibroblast-like morphology (30). Notably, CD105(+)/CD34 (–) fibroblasts exhibited higher expression of CD146 and CD106, markers associated with MSC identity, enhanced migratory capacity and immunomodulatory function (31–34). In this context, CD146 has been proposed as a marker of osteoprogenitor MSCs, particularly in BM–derived subpopulations, and has been associated with increased self-renewal, multipotency, and resistance to senescence (31–33). Similarly, CD106 has been implicated in tumor progression and bone metastasis through its interaction with integrins and its ability to promote osteoclast activation (35, 36). The enrichment of these markers suggests that CD105(+)/CD34(–) fibroblasts could represent a CAF subset with preserved mesenchymal progenitor-like features and potential relevance for bone-related metastatic processes. In line with this, both fibroblast subpopulations expressed classical CAF markers such as α-SMA and FAP (37, 38). CD105(+)/CD34(–) fibroblasts, however, displayed a more activated molecular profile, including increased expression of PDGFRα, desmin, and P4HB. These molecules have been associated with fibroblast activation, extracellular matrix remodeling, and tumor progression across multiple cancer types (39, 40).

Moreover, when examining self-renewal characteristics, both fibroblast subpopulations exhibited similar CFU-F frequencies, reflecting comparable cloning efficiency and indicating that both cell types retain stemness-related features. However, CD105(+)/CD34(–) fibroblasts formed colonies with a significantly higher number of stromal cells. This finding suggests a distinct colony architecture and supports a higher proliferative potential compared with CD105(–)/CD34(–) fibroblasts. This interpretation is supported by cell cycle analysis, which revealed a higher proportion of CD105(+)/CD34(–) fibroblasts in the S phase and a reduced fraction of cells in the G0/G1 phase, indicative of increased proliferative activity (41, 42). In addition, CD105(+)/CD34(–) fibroblasts displayed higher metabolic activity and cell viability, as assessed by the MTS assay, consistent with a more metabolically active and proliferative phenotype. These observations are consistent with our previous findings in primary tumors from patients with early IDC, where CD105 expression in CD34-negative, spindle-shaped stromal cells was associated with increased intratumoral stroma, higher fibroblast abundance, and greater degrees of desmoplasia (13). Collectively, these findings may support an association between CD105-expressing fibroblasts and stromal expansion as well as fibrotic features within the breast tumor microenvironment (13).

Oxidative stress emerged as another distinguishing feature of CD105(+)/CD34(–) CAFs, which exhibited elevated total and mitochondrial ROS levels. Increased ROS production in CAFs has been associated with hypoxia, metabolic reprogramming, and the secretion of pro-invasive factors that enhance tumor cell motility, angiogenesis, and metastatic potential (43–47). In this context, oxidative stress may trigger multiple cellular changes in stromal cells, including the activation of senescence-associated programs. Although both fibroblast subpopulations displayed comparable levels of cellular senescence, elevated oxidative stress may still contribute to the acquisition of a pro-tumorigenic secretory phenotype, as described for senescent CAFs in breast cancer (48, 49). Nevertheless, additional studies specifically addressing senescence-associated pathways will be required to fully substantiate these observations.

At a transcriptional level, CD105(+)/CD34(–) fibroblasts showed increased expression of genes related to mesenchymal-like identity, extracellular matrix remodeling, osteogenic differentiation, osteoclastogenesis regulation, migration and tumor progression. This transcriptional profile included elevated expression of MCAM (CD146), VCAM (CD106), TNC, RUNX2, and RANKL. TNC expression in CAFs has been associated with enhanced tumor aggressiveness, cancer stem cell signaling, and poor clinical outcomes in breast cancer (50–54). Similarly, elevated RUNX2 and RANKL expression has been reported in breast tumors with a propensity for bone involvement, where these factors are associated with tumor progression and stromal–tumor interactions rather than direct causal effects (55–61). Our previous findings demonstrating the co-expression of CD105 and receptor activator of NF-κB (RANK) in spindle-shaped stromal cells not associated with the vasculature in early primary IDC, together with their migratory response to RANKL, further support an association between this CAF subset and RANKL-related signaling pathways (62). Together, these observations are consistent with a potential involvement of CD105(+)/CD34(–) CAFs in stromal–tumor interactions linked to bone-associated tumor progression.

Beyond the characterization of the secretome, differential abundance patterns were observed between fibroblast subpopulations. CD105(+)/CD34(–) CAFs exhibited higher relative abundance of extracellular matrix–related proteins, including laminin and lumican, as well as Gal-3BP and the complement component C1s, whereas CD105(–)/CD34(–) CAFs showed higher abundance of type II keratins. Notably, proteins enriched in the CD105(+)/CD34(–) secretome have been previously associated with tumor invasion, metastasis, and prognosis in breast cancer (63–70). Overall, although both fibroblast subpopulations displayed secretory profiles consistent with activated, pro-tumoral CAF phenotypes, differences in the relative abundance of specific secreted proteins suggest heterogeneity in their stromal secretory signatures. The absence of expected cytokines such as IL-6, CCL-2, or RANKL, as well as a senescence-associated secretory profile, may reflect technical limitations inherent to mass spectrometry–based secretome analyses, including incubation time, protein dilution in culture media, and potential interference from media components.

Gene expression analyses revealed that CM from CD105(+)/CD34(–) fibroblasts induced the expression of stemness-associated genes, including SOX2 and OCT4, in MDA-MB231 cells. In addition, CM from this fibroblast subpopulation upregulated genes related to osteogenic differentiation, bone mineralization, and osteoclastogenesis—such as BMP2, BMP6, FGF10, RUNX2, and RANKL—in both MCF-7 and MDA-MB231 cell lines. These observations are consistent with, and may help explain, our previous findings linking CD105-positive CAFs to tumor microcalcifications and aggressive breast cancer phenotypes, as well as more recent evidence suggesting an association between anarchic tumor microcalcifications and poor prognosis, particularly in relation to bone metastasis (18, 71). Collectively, these data are compatible with a potential involvement of CD105(+)/CD34(–) CAFs in modulating tumor cell programs associated with stemness and bone-related features, which have been implicated in osteomimicry and tumor–stroma interactions relevant to breast cancer progression toward bone involvement (72–74). Nevertheless, further studies will be required to elucidate the molecular mechanisms underlying these effects and their implications for therapeutic strategies targeting tumor–stromal interactions.

Taken together, these observations underscore the importance of investigating the phenotypic, molecular, and functional heterogeneity of CAF subpopulations in breast tumors to improve prognostic assessment and inform the development of more precise therapeutic approaches. However, the coexistence of multiple CAF subtypes with distinct origins, markers, and functional properties within the breast tumor microenvironment represents a major challenge. In this context, our findings suggest that CD105(+)/CD34(–) CAFs may represent a biologically relevant stromal subset in breast cancer. Consequently, a more refined characterization and stratification of CAF subpopulations will be essential before the implementation of therapeutic strategies aimed at targeting tumor-promoting stromal components.

## 5 Limitations

This study has some limitations that should be taken into consideration. The relatively small sample size (BCPs, n = 10) may restrict the generalizability of the findings. In addition, although the biological effects of conditioned media on breast cancer cell lines were clearly demonstrated, the specific soluble factors responsible for these effects were not individually identified. In this context, the proteomic analysis was designed as an exploratory and hypothesis-generating approach, and targeted validation and mechanistic studies will be required in future work to define the functional contribution of individual secreted proteins. Despite these limitations, the consistency of the phenotypic, functional, and molecular alterations observed across samples supports the robustness of the conclusions.

## 6 Conclusion

CAFs represent a highly relevant and heterogeneous stromal population involved in breast cancer progression. These cells become activated at different stages of tumor development and influence tumor behavior through dynamic interactions with cancer cells and other components of the tumor microenvironment, including the extracellular matrix and immune cells. Despite their recognized importance, the molecular classification and functional specialization of CAF subpopulations in breast cancer remain incompletely defined, which currently limits their translational and clinical exploitation.

In this study, we characterized the phenotypic and functional features of two fibroblast subpopulations within the breast tumor microenvironment defined by CD105(+)/CD34(-) and CD105(-)/CD34(-) expression profiles. Our results demonstrate differential expression of markers associated with mesenchymal identity and fibroblast activation, including CD146, CD106, P4HB, and desmin, in CD105^+^/CD34^−^ fibroblasts. In parallel, this subpopulation exhibited distinct functional properties, such as increased proliferative activity, elevated intracellular ROS levels, and differential paracrine effects on breast cancer cell migration and proliferation. Collectively, these findings highlight functional heterogeneity among tumor-associated fibroblasts and underscore the existence of distinct stromal cell subsets within breast tumors. Gene expression analysis and secretome profiling further provided insight into potential differences in stromal–tumor communication between the two fibroblast subpopulations. Notably, CM derived from CD105(+)/CD34(-) fibroblasts induced the expression of stemness-associated and osteoblastic-related genes in MDA-MB-231 cells. Although these observations do not establish a direct mechanistic relationship, they are consistent with previous studies describing epithelial–mesenchymal plasticity and lineage reprogramming in aggressive breast cancer cells in response to microenvironmental cues. Taken together, these data extend previous work from our group and others, supporting the concept that CD105(+)/CD34(-) fibroblasts represent a functionally distinct CAF subset that may contribute to tumor progression–associated stromal signaling. More broadly, this study emphasizes the complexity of CAF subpopulations in breast cancer and provides a descriptive and functional framework for future mechanistic studies aimed at defining the precise roles of specific CAF subtypes and their potential relevance as therapeutic targets.

## Ethics approval

The study was conducted according to the guidelines of the Declaration of Helsinki. Furthermore, this research received approval from the Ethics Committee for Research Protocols of the Hospital Italiano in Buenos Aires, Argentina (approval: no5009).

## Data Availability

The data that support the findings of this study are not publicly available due to ethical restrictions, as they are derived from patient samples. Sharing these data with third parties could compromise patient confidentiality and privacy. For further inquiries, please contact the corresponding authors, María Belén Giorello, PhD, and Norma Alejandra Chasseing, PhD.

## Glossary

α-SMA: Alpha-Smooth Muscle Actin
BCPs: breast cancer patients
BMP: bone morphogenetic protein
CAFs: cancer associated fibroblasts
CD105: endoglin
CCL-2: chemokine (C-C motif) Ligand 2
CFU-F: colony forming units-fibroblastic
CM: conditioned media
COL3A1: collagen, type iii, alpha 1
ER: estrogen receptor
FAP: fibroblast activation protein
FBS: fetal bovine serum
FGF: fibroblast growth factor
Gal3-BP: galectin-3 binding ligand
Her2/neu: human epidermal growth factor receptor 2
IDC: invasive ductal breast cancer
IGFBP: insulin-like growth factor-binding protein
IL-6: interleukin 6
MCAM (CD146): melanoma cell adhesion molecule
MSCs: mesenchymal stem/stromal cells
OCT4: octamer-binding transcription factor 4
PDGFRα: platelet-derived growth factor receptor alpha
P4HB: prolyl 4-hydroxylase subunit beta
PR: progesterone receptor
RANKL: receptor activator of nuclear factor-kappa β
ROS: reactive oxygen species
RUNX2: runt-related transcription factor 2
SA-β-gal: senescence-associated β−galactosidase
SPARC: secreted protein acidic and rich in cysteine
SOX2: sry-box transcription factor 2
TGF-β: transforming growth factor beta
TNC: tenascin C
VCAM (CD106): vascular cell adhesion molecule
