# Supplementary figures and images for: Distinctive features of cancer-associated fibroblasts expressing CD105, a novel biomarker for bone metastasis, in early-stage invasive ductal breast cancer
Source: Front Endocrinol (Lausanne). 2026 Feb 20;17:1766643. doi: 10.3389/fendo.2026.1766643 (PMC12962955; doi:10.3389/fendo.2026.1766643)

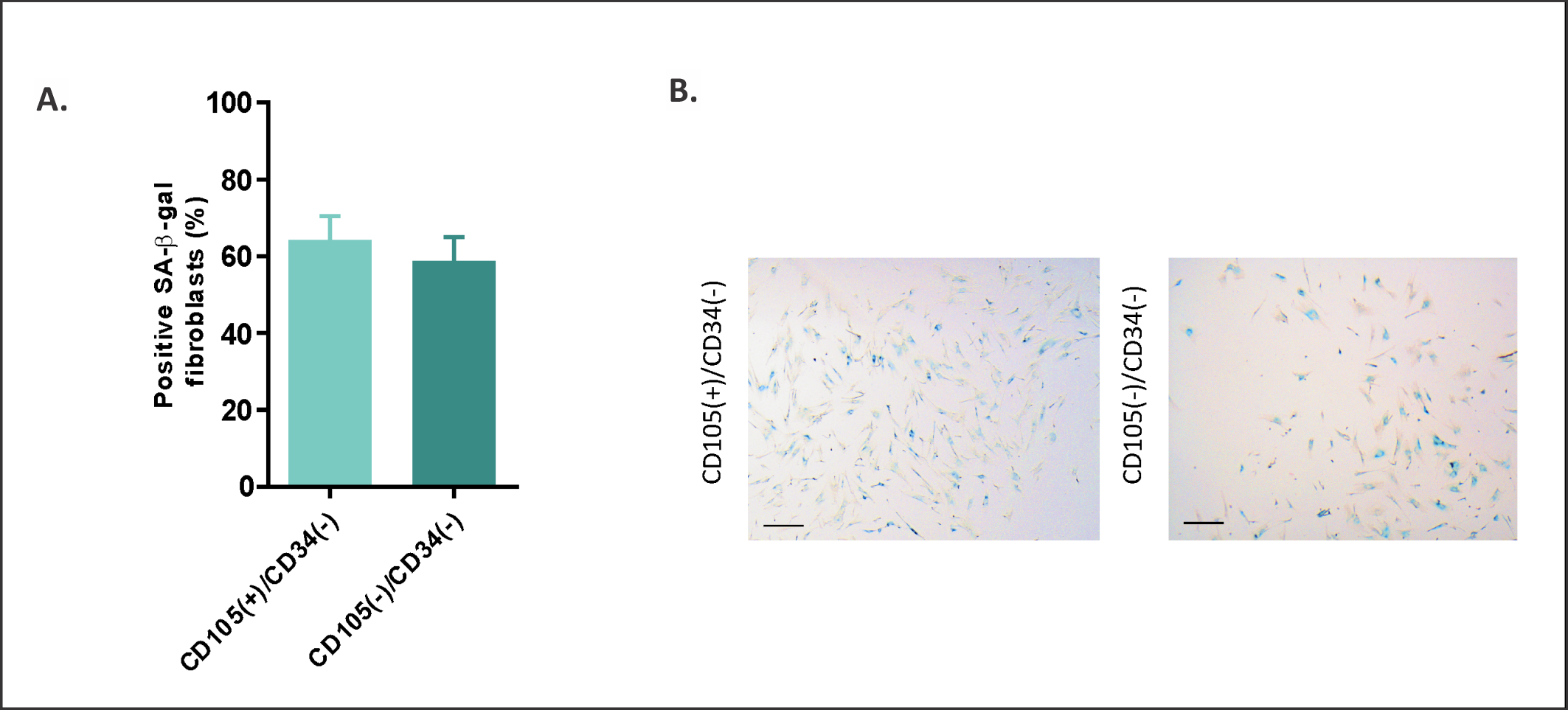

Supplement: Supplementary file 1 [file Image1.tif]
