# Supplementary material for: Distinctive features of cancer-associated fibroblasts expressing CD105, a novel biomarker for bone metastasis, in early-stage invasive ductal breast cancer
Source: Front Endocrinol (Lausanne). 2026 Feb 20;17:1766643. doi: 10.3389/fendo.2026.1766643 (PMC12962955; doi:10.3389/fendo.2026.1766643)
Supplement: Supplementary file 2 [file DataSheet1.docx]

Supplementary Material

# Supplementary Materials and Methods

## Isolation and Expansion of Fibroblasts from Primary Breast Tumor Tissue

Immediately after surgery, breast tumor tissues were placed in DMEM-F12 (cat. 12500-062, Gibco). Subsequently, they were washed with the same medium supplemented with an antibiotic-antimycotic solution (ATB/ATM) (cat.15240, Gibco), with a final concentration of 100 IU/ml of penicillin, 100 µg/ml of streptomycin, 25 µg/ml of amphotericin B, and 2 mM of L-glutamine added to the medium (hereafter referred to as supplemented). The tissue was cut with a scalpel in a tissue culture plate and treated with 0.1% collagenase type III /hyaluronidase (cat. 07912, StemCell, Vancouver, Canada) overnight at 37°C with gentle agitation. After this period, the sample was centrifuged at 40 g for 2 min at room temperature (RT). The resulting pellet, rich in organelles and undissociated tissue, was discarded. The obtained supernatant was transferred and centrifuged again at 100 g for 2 min at RT. The pellet obtained at this step is enriched in epithelial cells, while the supernatant is enriched in fibroblasts, the cell fraction of interest. A final centrifugation of the supernatant at 200 g for 5 min, at RT, was performed. The resulting pellet, rich in fibroblasts (like- cancer associated fibroblasts [CAFs]), was resuspended in α-minimal essential medium (α-MEM, cat. 11900024, Gibco) with the ATB/ATM solution, previously described (supplemented α-medium). Cell counting was performed using a 3% acetic acid solution in water, and the viability of CAFs was determined by the trypan blue exclusion test (0.04% in Phosphate-Buffered Saline, PBS). For primary cultures, 3.75 x 10^5^ cells/flask of 25 cm^2^ (cat. 5510100, Orange Scientific) were incubated in 10 ml of supplemented α-medium with the addition of 20% fetal bovine serum (FBS) (Natocor). After 24 hours, non-adherent cells were removed and the medium was renewed to leave only adherent cells. Incubations were carried out at 37°C, 5% CO_2_, and humidity. The medium was renewed every 7 days, and when the culture reached 70-80% confluence, adherent cells were treated with a trypsin- ethylenediaminetetraacetic acid (EDTA) solution (0.05%–0.02% in PBS, respectively, cat. 15400, Gibco) to detach them. The adherent cells obtained from this first subculture are predominantly differentiated stromal cells (especially CAFs). The cells were split to two flasks in order to increase the number of cells, which were incubated in supplemented α-medium with the addition of 20% FBS. The culture medium was renewed every 7 days until CAFs reached 70-80% confluence again. At that point, CAFs from the second subculture were trypsinized, and the separation of CD105(+)/CD34(-) and CD105(-)/CD34(-) fibroblast subpopulations was subsequently performed [1–3].

## Separation of CD105(+)/CD34(-) and CD105(-)/CD34(-) Fibroblasts

CAFs from the 2^nd^ subculture was centrifuged at 200 g for 5 min. The resulting pellet was suspended in buffer [PBS, 0.5% bovine serum albumin (BSA, cat. A7030, Sigma Aldrich), and 2 mM EDTA (0.1461 g/L, cat. 15400054, Gibco) at pH 7.2] plus anti-CD34-PE antibody (1/10, cat. 130-096-140, Biotechnologies Inc Magnetic Separation Kit, MACS MiltenyiBiotec) and incubated at 4°C in the dark for 10 min. Later, CAFs were washed and centrifuged at 200 g for 10 min. The pellet was suspended in the previously described buffer with microbeads (cat. 130-091-271, Biotechnologies Inc Magnetic Separation Kit, MACS MiltenyiBiotec). After incubating at 4°C for 15 min, CAFs were washed with the same buffer PBS and centrifuged again at 200 g for 10 min. The pellet was resuspended with buffer previously described and passed through the magnetic separation column. The eluate from the column was collected, as it contained the population of CD34(-) stromal cells of interest. This cellular fraction was centrifuged again at 200 g for 10 min, and the pellet was resuspended with described buffer, adding anti-CD105-PE antibody (1/10, cat. 130-096-906, Biotechnologies Inc Magnetic Separation Kit, MACS MiltenyiBiotec). The same steps previously described above for the anti-CD34-PE Antibody were repeated. Finally, the cells were passed through the magnetic separation column, where CD105(-)/CD34(-) CAFs that did not bind to the column were collected. The column was then removed from the magnet to collect CD105(+)/CD34(-) cells. Both cellular fractions were centrifuged at 200 g for 10 min and were cultured in 25 cm^2^ culture flasks (15,000 cells/cm^2^), which were incubated in supplemented α-medium with the addition of 20% FBS. The medium was renewed every 7 days, and when the culture reached 70-80% confluence, adherent cells were treated with a trypsin-EDTA solution (0.05%-0.02%, respectively, in PBS). At this point, CAFs from the 3^rd^ subculture after passage through the column were trypsinized and incubated at a concentration of 240 viable cells/cm^2^ (4^th^ subculture). Low cell density favors the growth of multipotential and high self-renewal MSCs and, therefore, fibroblasts derived from them. These 4^th^ subcultures were incubated for 12 days, with a medium renewal at day 6. After this 12-day period, we considered the obtained cells as fibroblasts derived from MSCs. To increase cell yield, CD105(+)/CD34(-) and CD105(-)/CD34(-) fibroblasts were plated at 3,000 viable cells/cm^2^ and maintained by changing the medium every 7 days until reaching 70-80% confluence (5^th^ subculture). With these cellular fractions, the rest of the assays were conducted. Finally, the conditioned media (CM) were collected from the last 48 hours (serum-free) of these last 70-80% confluent subcultures. For proteomic studies of these CM and to investigate their action on human breast cancer cells of the MCF-7 and MDA-MB231 cell lines, a pool was created with them.

## Analysis of CD105 and CD34 Expression in Cancer-Associated Fibroblasts from Paraffin-Embedded Breast Cancer Samples

The analysis of CD105 and CD34 expression in CAFs was conducted according to the immunohistochemistry methodology described by Giorello et al. [4]. The antibodies used for double staining were as follows: anti-CD105 primary human antibody (goat IgG; AF1097; R&D Systems) and anti-CD34 antibody (mouse IgG1; M7165; Dako). The following detection systems were used: for CD105, the LSAB+ System-HRP (K0690, Dako, Santa Clara, CA, United States) and 3–3′-diaminobenzidine (Liquid DAB+ Substrate Chromogen System; K3468, Dako, Santa Clara, CA, United States); for CD34, the Vectastain ABC-Alkaline Phosphatase kit (Ak-5000; Vector Laboratories) and the Vector Red Substrate Kit (SK-5100; Vector Laboratories), both following the manufacturer’s instructions. Negative controls were conducted by incubating tissue sections without primary antibodies, along with irrelevant goat IgG (AB-108-C; R&D Systems) and mouse IgG1 (MAB002; R&D Systems). Duplicate assays were performed for each sample.

## Cell Culture Protocol for Breast Cancer Cell Lines

We utilized two human breast cancer cell lines: MCF-7 and MDA-MB231, obtained from the American Type Culture Collection (USA). These cells were cultured in DMEM/F12 medium supplemented with phenol red, which contained 100 IU/ml penicillin, 100 µg/ml streptomycin, 25 µg/ml amphotericin B, and 2 mM L-glutamine (referred to us as supplemented DMEM/F12). Additionally, MCF-7 cells were cultured with 2 µg/ml humanized pig insulin from Beta Laboratory, Argentina. All cell cultures were maintained in a 10% FBS environment, with a cell density of 4 x 10^4^ viable cells/cm^2^. The medium was renewed every three days. Cells were incubated at 37°C in a 5% CO_2_-humidified environment until reaching confluence. Upon confluence, adherent cells were washed with PBS and detached using trypsin-EDTA solution. Cell viability was assessed using the 0.04% trypan blue dye exclusion method. Aliquots of each cell line suspension were cryopreserved under nitrogen until required for subsequent assays. For experiments involving migration, proliferation and gene expression of cells from MCF-7 and MDA-MB231 lines were used up to the 4^th^ subculture [5].

## Study of the Phenotype of CD105(+)/CD34(-) and CD105(-)/CD34(-) Fibroblastic Subpopulations

A total of 3 x 10^5^ cells from CD105(+)/CD34(-) and CD105(-)/CD34(-) subpopulation fractions were used per reaction tube, which were centrifuged at 200 g for 5 min. Subsequently, cells were resuspended in PBS with 1% BSA. Next, they were incubated with specific monoclonal antibodies conjugated with different fluorochromes against the following human antigens for 30 minutes at RT: CD105 (cat. FAB10971V, R&D Systems Inc), CD34 (cat. FAB7227P, R&D Systems Inc), CD90 (cat. FAB2067G, R&D Systems Inc), CD73 (cat. FAB5795A, R&D Systems Inc), α-SMA (cat. IC1420A, R&D Systems Inc), FAP (cat. FAB3715P, R&D Systems Inc), P4HB (cat. ab137110, Abcam), desmin (cat. ab32362, Abcam), TNC (cat. ab215369, Abcam), CD146 (cat. AF932, R&D Systems Inc), CD106 (cat. BBA19, R&D Systems Inc), PDGFRα (cat. FAB1264P, R&D Systems Inc), CD19 (cat. FAB4867A, R&D Systems Inc), CD11b (cat. FAB1699V, R&D Systems Inc), and CD14 (cat. FAB3832P, R&D Systems Inc). Isotype controls (cat. IC002V, cat. IC003G, cat. IC0041A, cat. IC002A, cat. IC002P, R&D Systems Inc,) were run in parallel using the same concentration of each antibody tested. For unconjugated primary antibodies, Cy™5 AffiniPure™ Goat Anti-Rabbit IgG (cat. 111-175-144, Jackson ImmunoResearch) and Alexa Fluor® 488 AffiniPure™ Donkey Anti-Goat IgG (cat. 705-545-147, Jackson ImmunoResearch, West Baltimore Pike, USA) were used as fluorophore-conjugated secondary antibodies. The concentrations recommended by the manufacturers for each antibody were used in flow cytometry analysis. After incubation, cells from each tube were washed twice with 1% PBS-BSA and centrifuged at 200 g for 4 min in each wash. Finally, cells were resuspended in PBS and analyzed for 10,000 events in each case by flow cytometry (FACScanto II, BD Biosciences). For intracellular markers, cells were fixed in 4% paraformaldehyde (158127, Sigma-Aldrich) for 20 minutes at 4°C. After a wash in PBS, cells were incubated with the antibody cocktail and supplemented with 0.1% saponin (Sigma-Aldrich, #S7900) for 45 minutes at RT. FlowJo software was used to analyze the data, using isotype controls to accurately position analysis quadrants and obtain relative fluorescence indices (RFI: specific surface molecule fluorescence index/specific isotype control fluorescence index). Experiments were performed in duplicate using different fibroblast preparations from breast cancer samples obtained from 10 patients.

## Study of Self-Renewal, CFU-F assay and Morphology of Stromal Cells within Colonies

A total of 2,500 cells from CD105(+)/CD34(-) and CD105(-)/CD34(-) subpopulation fractions were cultured per 25 cm^2^ flask (100 viable cells/cm2) in supplemented α-medium and 20% FBS, at 37°C, 5% CO_2_, in a humidified environment for 7 days. Adherent fibroblasts were then washed with PBS and incubated again with fresh medium for an additional 7 days. After 14 days, the flasks were washed with PBS, air-dried, and fixed with 100% methanol (cat. 2000165508, Biopack, Argentina) for 15 minutes at RT. Subsequently, CFU-Fs (colony forming unit-fibroblastics) were stained with pure Giemsa (cat. 48900 Sigma Aldrich) for 5 minutes at RT, then washed with water and air-dried. Colonies with 50 cells or more were scored as CFU-Fs under an inverted light microscope at 40X total magnification. The frequency of CFU-Fs is indicated by colony forming efficiency, defined as the number of CFU-Fs obtained for every 2,500 fibroblasts seeded. For efficiency evaluation, CFU-F assays were performed in duplicate on fibroblast preparations obtained from breast cancer samples from 10 patients. Additionally, the stromal cell density per optical field of CFU-Fs was determined [6]. To achieve this, ten pictures were captured from different optical fields of each CFU-F culture. Furthermore, an evaluation of morphological changes was conducted in stromal cells exhibiting fibroblast-like characteristics within CFU-F cultures. The analysis included measuring the area, longitudinal axis of the ellipse, and horizontal axis using three pictures obtained from three typical regions (three optical fields, images at 200X magnification) of each CFU-F culture, with 10 cells per image. These measurements were analyzed using FIJI software [7].

## Assessment of Cell Viability as an Indirect Measure of Proliferation Capacity

A total of 1 x 10^4^ cells/well from CD105(+)/CD34(-) and CD105(-)/CD34(-) subpopulation fractions were cultured in a 96-well plate (cat. 4430100, Orange Scientific) for 24 hours in supplemented α-MEM medium without phenol red (cat. 41061029, Gibco) and in the presence of 20% FBS at 37°C, 5% CO_2_, and humidity. After this period of plastic adherence, cells were washed with 1X PBS, and cell arrest was induced by an additional 48-hour culture in supplemented α-MEM medium without phenol red and without FBS. Subsequently, another wash with 1X PBS was carried out, and the stimulation stage took place, where cells were cultured for 48 hours in supplemented α-MEM medium without phenol red with 5% FBS at 37°C, 5% CO_2_, and humidity. Basal controls were performed with supplemented α-MEM medium without phenol red without FBS. Cell proliferation was evaluated using the CellTiter 96® AQueous One Solution kit (cat. G5421, Promega). This assay is based on a colorimetric method that determines the number of viable proliferating cells by reducing the tetrazolium compound [3-(4,5-dimethylthiazol-2-yl)-5-(3-carboxymethoxyphenyl)-2-(4-sulfophenyl)-2H-tetrazolium, inner salt; MTS] to a colored product called formazan. This conversion occurs due to the activity of dehydrogenase enzymes in metabolically active cells, using NADPH or NADH. For the assays, the CellTiter 96® AQueous One Solution reagent was added directly to the culture wells, incubated for 1-4 hours at 37°C, 5% CO_2_, and controlled humidity, and then the absorbance at 490 nm was recorded using a 96-well plate reader. The amount of formazan product generated is directly proportional to the number of living cells in culture. The optical density (OD) value of each sample under study was obtained by subtracting the control OD (supplemented α-MEM medium without phenol red and FBS) from its respective sample value. All experiments were performed in triplicate. Therefore, studying the number of viable cells allows us to indirectly infer cell proliferation, as higher cell viability typically reflects increased proliferation.

## Study of Cell Cycle

For the study of cell cycle, cells were resuspended at a concentration of 2 x 10^6^ cells/ml from CD105(+)/CD34(-) and CD105(-)/CD34(-) subpopulation fractions in 1X PBS and fixed in cold methanol. The fixed cells were centrifuged at 200 g for 10 min. Subsequently, they were incubated with 200 mg RNase A in 1X PBS (#GE101-01, Beijing TransGen Biotech Co.,) for 20 min at 37 °C. The cells were centrifuged again at 200 g for 10 min and incubated with 20 mg propidium iodide in 1X PBS (PI, Cat. 81845, Sigma Aldrich) at RT in the dark for 30 min, inverting the tube every 10 min. Finally, centrifugation at 200 g for 10 min was performed, and the cells were resuspended in 1X PBS. Samples were analyzed using a FACScanto II (Becton Dickinson). Cell cycle analysis was determined using FlowJo (v. X). Experiments were repeated twice for each sample.

## Gene Expression Study

### RNA Extraction and cDNA Synthesis

The total RNA from the CD105(+)/CD34(-) and CD105(-)/CD34(-) subpopulations of the 5^th^ subculture was isolated using TRI Reagent® (cat. TR 118 Molecular Research Center, Inc) "and quantified using the NanoDrop™ 2000/2000c spectrophotometer (cat. ND-2000, ThermoScientific). Starting with 1 µg of RNA per sample, cDNA was obtained using the High-Capacity cDNA Reverse Transcription Kit (cat. 4368814, Applied Biosystems, Foster City, CA, USA) with random primers, following the manufacturer's recommendations.

### Quantitative Real-time PCR

Samples were analyzed using the FS UNIVERSAL SYBR GREEN MASTER ROX master mix (cat. 04913850001, ROCHE) in a CFX96TM TOUCH REAL-TIME PCR system (Bio-Rad, Hercules) under standard amplification conditions, followed by a melting curve analysis. Cycle threshold values were normalized using the glyceraldehyde-3-phosphate dehydrogenase (GAPDH) reference gene. Primer sequences are provided in Supplementary Table 1. The expression of stemness genes (self-renewal and multipotentiality, particularly osteoblastic differentiation), as well as genes related to osteoclastogenesis regulation, migration capacity, and tumor evolution, were studied [octamer-binding transcription factor 4 (OCT4), sry-box transcription factor 2 (SOX2), melanoma cell adhesion molecule (MCAM or CD146), vascular cell adhesion molecule (VCAM or CD106), bone morphogenetic protein (BMP-6), runt-related transcription factor (RUNX-2), chemokine (C-C motif) Ligand 2 (CCL-2), receptor activator of nuclear factor-kappa β ligand (RANKL), interleukin 6 (IL-6), tenascin C (TNC)]. Experiments were conducted in duplicate for each sample.

## Study of Oxidative Stress [Mitochondrial ROS (superoxide anion) and Total ROS]

A total of 2 x 10^5^ cells from CD105(+)/CD34(-) and CD105(-)/CD34(-) subpopulations were centrifuged at 200 g for 10 min at RT. Subsequently, they were resuspended in 200 µl of 1X PBS and centrifuged again at 200 g for 10 min. Levels of total and mitochondrial reactive oxygen species (ROS) were detected using the fluorescent dyes CellROX Oxidative Stress Reagents (cat. C10444, Thermo Fisher) and MitoSOXTM Red (cat. M36008, Molecular Probes,), respectively. The two cellular fractions were incubated with 250 µl of CellROX or 500 µl of MitoSOXTM for 30 min at 37°C in the dark. Subsequently, they were centrifuged at 200 g for 10 min and resuspended in 400 µl of 1X PBS. Cells were incubated for 5 min with 5 μg/ml 4′,6-diamidino-2-phenylindole (DAPI, cat. D9542, Sigma Aldrich) at RT in the dark. Finally, they were centrifuged at 200 g for 10 min and resuspended in 400 µl of 1X PBS. Fluorescent intensity was analyzed by flow cytometry (FACScanto II; Becton Dickinson), selecting the mean fluorescence intensity in viable cells (DAPI negative) in the corresponding fluorescence channel (FITC for CellROX and PE for MitoSOXTM). Therefore, the fluorescence intensity of the MitoSOX probe (excitation λ: 510 nm; emission λ: 580 nm) was evaluated in the red channel, that of the CellROX probe (excitation λ: 508 nm; emission λ: 527 nm) in the green channel, only in viable cells, selected as DAPI-negative cells (excitation λ: 340 nm; emission λ: 461 nm), in the blue channel. The results were analyzed using FlowJo software (v. X, Tree Star, Inc., Ashland, OR, USA). Experiments were repeated twice for each sample.

## Study of Cellular Senescence

A seeding of 1 x 10^4^ cells/well, derived from CD105(+)/CD34(-) and CD105(-)/CD34(-) subpopulations, was performed in 24-well plates (cat. 4430300, Orange Scientific). After 24 hours, cultures were washed with PBS and further incubated for 72 hours in supplemented α-MEM medium with the addition of 20% FBS at 37°C, 5% CO_2_, and humidity. Subsequently, cells were washed with PBS and fixed with a solution containing 37% vol/vol formaldehyde (cat. 47608, Sigma Aldrich), 25% vol/vol glutaraldehyde (cat. G7651, Sigma Aldrich), and 1X PBS for 5 min at RT. Additional washes with 1X PBS were carried out, and cells were incubated for 16 hours at 37°C without CO_2_ with a fresh senescence-associated β‑galactosidase (SA-β-gal) staining solution [1 mg/mL 5-bromo-4-chloro-3-indolyl β-D-galactopyranoside (X-Gal) 150 mM NaCl, 2 mM MgCl_2_, 40 mM citric acid/sodium phosphate buffer (pH 6), 5 mM potassium ferrocyanide, and 5 mM potassium ferricyanide, cat. 11680293001, Sigma Aldrich]. Stained cells were visualized and photographed using an Olympus CKX41 inverted microscope (Olympus, Shinjuku-ku, Tokyo, Japan) with a 20X/0.4 objective and Olympus Q-Color 5 camera. Experiments were repeated twice for each sample.

## Secretome study

For the following assay, a pool of CM samples from BCPs (n = 10) collected at 48 hours from the culture of both subpopulations in the absence of FBS was used. These CM were lyophilized to concentrate the secreted factors by both cellular fractions. Subsequently, the samples underwent 1D electrophoresis using denaturing 10% polyacrylamide gels under reducing conditions, with a load of 30 µg of proteins per lane. The run was carried out for 60 min at a constant voltage of 100 V until the running front was positioned 1 cm below the stacking gel. Immediately after the run, the gels were incubated with a fixing buffer consisting of 50% v/v methanol and 2% v/v phosphoric acid (cat. 93752 , Sigma Aldrich) in sterile water (cat. 956-A, Rivero), agitating for 5 hours at RT. Following this, three washes were performed with ultra-pure water, and the gels were incubated with a balancing buffer composed of 33% v/v methanol, 17% p/v ammonium sulfate (cat. AX1385, Sigma Aldrich), and 3% v/v phosphoric acid in sterile water, agitating for 1 hour at RT. Finally, Coomassie Colloidal (cat. 1610803, BioRad) was added to the same balancing buffer at a final concentration of 0.066%, and the gels were incubated overnight with agitation at RT.

To destain the gels, three washes with ultra-pure water were performed, and polyacrylamide bands (each containing a protein band corresponding to the secretome of each fibroblast subpopulation) were cut using a sterile scalpel. The study was conducted in triplicate. The proteomics analysis was carried out by the Centro de Estudios Químicos y Biológicos por Espectrometría de Masa (CEQUIBIEM) – Facultad de Ciencias Exactas y Naturales – UBA, through the LFQ proteomic method (Label-free protein quantification). Patterns were obtained from the analysis on an HESI-Orbitrap Q Extractive mass spectrometer (Thermo Fisher). The raw spectra obtained were analyzed using Proteome Discoverer software (Thermo Scientific, v. 2.2) for protein identification. Based on the raw mass spectrometry data, the mass spectrometry service provided a report per group indicating the proteins identified for that group and the peptides used for such identification, using the Uniprot database. Statistically significant differences in relative abundances between the two analyzed groups [CD105(+)/CD34(-) vs CD105(-)/CD34(-) fibroblast] were sought using Perseus software (v2.0.11, available at https://maxquant.net/perseus/). Expressed proteins were organized into networks based on their associations using the online String software (available at <https://string-db.org/>).

## Effect of CM pool from CD105(+)/CD34(-) and CD105(-)/CD34(-) Fibroblastic Subpopulations on Breast Cancer Cells of the MCF-7 and MDA-MB231 Cell Lines

### Migration of Breast Cancer Cells

To evaluate cellular migration capability, we employed the wound healing assay, where wound closure served as an indicator of cellular motility. This technique involved creating multiple scratches or "wounds" on fully confluent cell monolayers of MCF-7 and MDA-MB231 cell lines. Following wound creation, the medium was completely replaced to eliminate any cellular debris. For the assay, a pool of CM samples from BCPs (n = 10) collected at 48 hours from the culture of both fibroblast subpopulations without FBS was utilized. Subsequently, cultures were exposed to different CM from CD105(+)/CD34(-) and CD105(-)/CD34(-) fibroblasts, as well as supplemented α-MEM with 10% FBS (positive control) and supplemented α-MEM alone (basal control). Quantification of cellular migration was performed by measuring the variation in wound width between time 0 (T0) and 12 hours (T12). Image areas were calculated using ImageJ software, determining the percentage of area occupied relative to the original wound (T0). Each assay was conducted in duplicate using pure CM, and the experiment was repeated 7 times.

### Assessment of Cell Viability as an Indirect Measure of Proliferation Capacity of Breast Cancer Cells

The same protocol used for fibroblasts was applied to breast cancer cells. In summary, 5 x 10^3^ breast cancer cells from MCF-7 or MDA-MB231 were cultured in 96-well plates under adherent conditions for 24 hours and arrest conditions for 48 hours at 37°C, 5% CO_2_, and humidified. The culture medium consisted of supplemented DMEM/F12 without phenol red, with 10% FBS for adherent conditions and without FBS for arrest conditions. Following this incubation period, the cells were further incubated for 48 hours with the following treatments: i) 100% CM pooled from CD105(+)/CD34(-) CAFs, ii) CD105(-)/CD34(-) CAFs, iii) 100% supplemented α-MEM + 5% FBS (positive control), and iv) 100% supplemented α-MEM (basal control, incubated for 48 hours at 37°C, 5% CO_2_, and humidified). Subsequent steps were carried out as previously described. Finally, the OD value of each sample was obtained by subtracting the OD value of the basal control from its respective sample value. All experiments were performed in triplicate, and the assay was repeated four times.

### Gene Expression in Breast Cancer Cells

The same procedure was performed for RNA extraction and subsequent reverse transcription to cDNA in the MCF-7 and MDA MB-231 cell lines, followed by Quantitative Real-time PCR. Prior to RNA extraction, the cell lines were treated for 48 hours with the CM pool corresponding to CD105(+)/CD34(-) CAFs, CD105(-)/CD34(-) CAFs, and supplemented α-MEM alone (as a basal control). The expression of stemness genes, focusing on self-renewal and multipotentiality, particularly osteoblastic differentiation, was studied (including OCT-4, SOX2, BMP2, BMP-6, fibroblast growth factor 10 (FGF10), RUNX-2 and RANKL; see supplementary Table 1). Experiments were conducted in duplicate for each sample, and the assay was repeated 7 times.

# Supplementary Results

## Cellular Senescence through SA-β-galactosidase Detection in CD105(+)/CD34(-) and CD105(-)/CD34(-) Fibroblastic Subpopulations

## Senescent CAFs within the tumor microenvironment play a crucial role in cancer progression by secreting pro-tumor factors [8,9]. Additionally, therapy-induced senescence in fibroblasts often results in resistance to chemo- and radiotherapy and stimulates the promotion of a pro-tumoral secretory phenotype [8,9]. The senescence program in these cells can be triggered by various stress factors, such as oxidative stress. Both CD105(+)/CD34(-) and CD105(-)/CD34(-) fibroblasts showed a comparable percentage of cells positive for SA-β-galactosidase, indicating a similar level of cellular senescence (Figure 3D and E).

# Supplementary Table 1 Primers

| Table 1. | |
| --- | --- |
| Primer | Sequence (5’-3’) |
| GAPDH-forward | CCACATCGCTCAGACACCAT |
| GAPDH-reverse | CATGGGTGGAATCATATTGGA |
| SOX2-forward | AGCTACAGCATGATGCAGGA |
| SOX2-reverse | GGTCATGGAGTTGTACTGCA |
| OCT4-forward | AGCGAACCAGTATCGAGAAC |
| OCT4-reverse | TTACAGAACCACACTCGGAC |
| MCAM -forward | TGAGGAGGTCGCTACCTGTGT |
| MCAM -reverse | GACTCCACAGTCTGGGACGA |
| VCAM -forward | GGG AAG ATG GTC GTG ATC CTT |
| VCAM -reverse | TCT GGG GTG GTC TCG ATT TTA |
| BMP2-forward | TCCATGTGGACGCTCTTTCA |
| BMP2-reverse | GGTCGACCTTTAGGAGACCG |
| BMP6-forward | TTGTGAACCTGGTGGAGTACG |
| BMP6-reverse | TCACCCTCAGGAATCTGGGAT |
| BMP7-forward | GCTTCGTCAACCTCGTGGAA |
| BMP7-reverse | AACCGGAACTCTCGATGGTG |
| FGF10-forward | GTGCGGAGCTACAATCACCT |
| FGF10-reverse | GCTGACCTTCCCGTTCTTCT |
| RUNX2-forward | CACAAGTGCGGTGCAAACTT |
| RUNX2-reverse | GGTAGTGACCTGCGGAGATT |
| CCL-2 -forward | GAAAGTCTCTGCCGCCCTT |
| CCL-2 -reverse | GGCATTGATTGCATCTGGCTG |
| IL-6-forward | TTCCAAAGATGTAGCCGCCC |
| IL-6-reverse | CTGAGATGCCGTCGAGGATG |
| RANKL-forward | AAACAGGCCTTTCAAGGAGC |
| RANKL-reverse | ACCATCGCTTTCTCTGCTCT |
| TNC-forward | TCTTGAAGGCAGGCGCAAAC |
| TNC-reverse | CCAAATGCCCAGGTGTGGACCGAT |

**Table 1.** *DNA primer sequences utilized in the study.*

# Supplementary Figure Legends

**Supplementary figure 1. Cellular senescent associated β galactosidase (SAβ gal) in CD105(+)/CD34(-) and CD105(-)/CD34(-) fibroblastic subpopulation isolated from the primary tumor of breast cancer patients (BCPs).** A. Staining assay for β‑galactosidase (SA-β‑gal). Presence of senescent CD105(+)/CD34(-) and CD105(-)/CD34(-) fibroblasts from BCPs (n=10). Values are expressed as mean ± SEM. Statistical analysis: unpaired t-test with Welch correction (p=0.5402). B. Representative image of CD105(+)/CD34(-) fibroblasts (left panel) and CD105(-)/CD34(-) fibroblasts (right panel) (100X). Senescent fibroblasts were stained blue. The scale bar represents 200 µm.

# Supplementary references

1. Kashyap D, Bhattacharya S, Irinike S, Khare S, Das A. Cancer associated fibroblasts modulate the cytotoxicity of anti-cancer drugs in breast cancer : An in vitro study. 2024;43:25–36.

2. Zhang K, Guo L, Li X, Hu Y, Luo N. Cancer-associated fibroblasts promote doxorubicin resistance in triple-negative breast cancer through enhancing ZFP64 histone lactylation to regulate ferroptosis. 2025;3.

3. Huang M, Li Y, Zhang H, Nan F. Breast cancer stromal fibroblasts promote the generation of CD44 + CD24 - cells through SDF-1 / CXCR4 interaction. 2010;1–10.

4. Giorello MB, Martinez LM, Borzone FR, Padin M del R, Mora MF, Sevic I, et al. CD105 expression in cancer-associated fibroblasts: a biomarker for bone metastasis in early invasive ductal breast cancer patients. Front Cell Dev Biol. 2023;11(August):1–16.

5. Martinez LM, Vallone VBF, Labovsky V, Choi H, Hofer EL, Feldman L, et al. Changes in the peripheral blood and bone marrow from untreated advanced breast cancer patients that are associated with the establishment of bone metastases. Clin Exp Metastasis. 2014;31(2):213–32.

6. Schindelin J, Arganda-Carreras I, Frise E, Kaynig V, Longair M, Pietzsch T, et al. Fiji: An open-source platform for biological-image analysis. Nat Methods. 2012;9(7):676–82.

7. Hofer EL, Labovsky V, La Russa V, Vallone VF, Honegger AE, Belloc CG, et al. Mesenchymal stromal cells, colony-forming unit fibroblasts, from bone marrow of untreated advanced breast and lung cancer patients suppress fibroblast colony formation from healthy marrow. Stem Cells Dev. 2010;19(3):359–69.

8. Gabai Y, Assouline B, Ben-Porath I. Senescent stromal cells: roles in the tumor microenvironment. Trends in Cancer. 2023;9(1):28–41.

9. Takasugi M, Yoshida Y, Ohtani N. Cellular senescence and the tumour microenvironment. Mol Oncol. 2022;16(18):3333–51.
